# Monitoring monocyte HLA-DR expression and CD4 + T lymphocyte count in dexamethasone-treated severe COVID-19 patients

**DOI:** 10.1186/s13613-024-01310-5

**Published:** 2024-05-18

**Authors:** Guillaume Monneret, Nicolas Voirin, Jean-Christophe Richard, Martin Cour, Thomas Rimmelé, Lorna Garnier, Hodane Yonis, Remy Coudereau, Morgane Gossez, Christophe Malcus, Florent Wallet, Marie-Charlotte Delignette, Frederic Dailler, Marielle Buisson, Laurent Argaud, Anne-Claire Lukaszewicz, Fabienne Venet, for the RICO study group, Remi Pescarmona, Christine Lombard, Magali Perret, Marine Villard, Marie Groussaud, Laetitia Itah, Inesse Boussaha, Françoise Poitevin-Later, Marie Simon, Auguste Dargent, Pierre-Jean Bertrand, Neven Stevic, Marion Provent, Laurie Bignet, Valérie Cerro, Laurent Bitker, Mehdi Mezidi, Loredana Baboi

**Affiliations:** 1 Immunology Laboratory, Hospices Civils de Lyon, Edouard Herriot Hospital, 5 Place d’Arsonval, 69437 Lyon, France; 2 EA 7426 “Pathophysiology of Injury-Induced Immunosuppression”, Joint Research Unit HCL-bioMérieux, (Université Claude Bernard Lyon 1 - Hospices Civils de Lyon - bioMérieux), 69003 Lyon, France; 3 Epimod, 01240 Dompierre Sur Veyle, France; 4 Medical Intensive Care Department, Hospices Civils de Lyon, Croix-Rousse University Hospital, 69004 Lyon, France; 5 Medical Intensive Care Department, Hospices Civils de Lyon, Edouard Herriot Hospital, 69437 Lyon, France; 6 Anesthesia and Critical Care Medicine Department, Hospices Civils de Lyon, Edouard Herriot Hospital, 69437 Lyon, France; 7 Immunology Laboratory, Hospices Civils de Lyon, Lyon-Sud University Hospital, 69495 Pierre Bénite, France; 8 Centre International de Recherche en Infectiologie (CIRI), Inserm U1111, CNRS, UMR5308, Ecole Normale Supérieure de Lyon, Université Claude, Bernard-Lyon 1, Lyon, France; 9 Intensive Care Department, Hospices Civils de Lyon, Lyon-Sud University Hospital, 69495 Pierre-Bénite, France; 10 Anesthesia and Critical Care Medicine Department, Hospices Civils de Lyon, Croix-Rousse University Hospital, 69004 Lyon, France; 11 Neurological Anesthesiology and Intensive Care Department, Hospices Civils de Lyon, Pierre Wertheimer Hospital, Lyon, France; 12 Centre d’Investigation Clinique de Lyon (CIC 1407 Inserm), Hospices Civils de Lyon, Lyon, France

**Keywords:** Immunomonitoring, Monocyte, HLA-DR, CD4, COVID-19, Dexamethasone, Sepsis

## Abstract

**Background:**

A 10-day dexamethasone regimen has emerged as the internationally adopted standard-of-care for severe COVID-19 patients. However, the immune response triggered by SARS-CoV-2 infection remains a complex and dynamic phenomenon, leading to various immune profiles and trajectories. The immune status of severe COVID-19 patients following complete dexamethasone treatment has yet to be thoroughly documented.

**Results:**

To analyze monocyte HLA-DR expression (mHLA-DR) and CD4 + T lymphocyte count (CD4) in critically ill COVID-19 patients after a dexamethasone course and evaluate their association with 28-day ICU mortality, adult COVID-19 patients (n = 176) with an ICU length of stay of at least 10 days and under dexamethasone treatment were included. Associations between each biomarker value (or in combination) measured at day 10 after ICU admission and 28-day mortality in ICU were evaluated. At day 10, the majority of patients presented decreased values of both parameters. A significant association between low mHLA-DR and 28-day mortality was observed. This association remained significant in a multivariate analysis including age, comorbidities or pre-existing immunosuppression (adjusted Hazard ratio (aHR) = 2.86 [1.30–6.32], p = 0.009). Similar results were obtained with decreased CD4 + T cell count (aHR = 2.10 [1.09–4.04], p = 0.027). When combining these biomarkers, patients with both decreased mHLA-DR and low CD4 presented with an independent and significant elevated risk of 28-day mortality (i.e., 60%, aHR = 4.83 (1.72–13.57), p = 0.001).

**Conclusions:**

By using standardized immunomonitoring tools available in clinical practice, it is possible to identify a subgroup of patients at high risk of mortality at the end of a 10-day dexamethasone treatment. This emphasizes the significance of integrating immune monitoring into the surveillance of intensive care patients in order to guide further immumodulation approaches.

**Supplementary Information:**

The online version contains supplementary material available at 10.1186/s13613-024-01310-5.

## Background

The pathophysiology of severe COVID-19 involves a complex interplay between viral invasion, activated local immune response, and systemic inflammatory processes [1]. In some individuals, an overactive inflammatory response can occur and contribute to tissue damage and organ dysfunctions [1]. In this context, mitigating the harmful effects of exacerbated inflammatory response has rapidly been hypothesized as a therapeutic approach in severe cases of COVID-19. Of available molecules, dexamethasone has emerged as an important option, particularly for individuals with respiratory complications. Its effectiveness in treating severe COVID-19 cases was demonstrated in the RECOVERY trial, a large-scale clinical trial conducted in the United Kingdom [2]. The trial found that dexamethasone reduced the risk of death by one-third in patients receiving mechanical ventilation and by one-fifth in those receiving oxygen support. Since the publication of the RECOVERY trial, 10-day dexamethasone treatment has been globally adopted as the standard-of-care for the treatment of severe COVID-19 patients [3–5].

After completing this beneficial 10-day dexamethasone treatment, there remains a challenge in clinical decision-making to guide further immunomodulation of patients, whether it be pro- or anti-inflammatory, owing to the complexity of the disease phenotypes and patient heterogeneity [6]. In this context, immune monitoring approaches could help in the formulation of subsequent therapeutic approaches [6]. Yet, as of our current knowledge, there has been no specific report on the immune status of a large cohort of severe COVID-19 patients who underwent a complete 10-day dexamethasone treatment.

Wit this in mind, the primary objective of this study was to analyze immune parameters measured in critically ill COVID-19 patients after a dexamethasone course in ICU and evaluate their association with 28-day mortality. We focused on CD4 + T lymphocyte count and monocyte HLA-DR (mHLA-DR) expression (utilizing standardized units), both robust biomarkers which deregulation has been largely described in bacterial sepsis [7, 8] and putatively available in routine care.

## Patients, material and methods

### Clinical study design, patient population and approval

Between March 2020 and May 2022, critically ill patients admitted to five ICUs from Lyon academic hospitals (Hospices Civils de Lyon, Lyon, France) who presented with pulmonary infection with SARS-CoV-2 confirmed by RT-PCR testing were prospectively included in the RICO (REA-IMMUNO-COVID) clinical study. The RICO study was approved by ethics committee (Comité de Protection des Personnes Ile de France 1—N°IRB / IORG #: IORG0009918) under agreement number 2020-A01079-30. This clinical study was registered at ClinicalTrials.gov (NCT04392401). This was an observational study that did not involve any specific procedures other than routine blood sampling. Oral information and non-opposition to inclusion in the study were mandatory and were systematically obtained before any blood sample was drawn. This was recorded in patients’ clinical files. If a patient was unable to consent directly, non-opposition was obtained from the patient’s legally authorized representative and reconfirmed from the patient at the earliest opportunity. Inclusion criteria were: (i) man or woman aged 18 or over, (ii) hospitalization in ICU for SARS-CoV-2 pneumopathy, (iii) first hospitalization in ICU for SARS-CoV-2 infection, (iv) diagnosis of SARS-CoV-2 infection performed by PCR in at least one respiratory sample, (v) sampling in the first 24 h after admission to ICU (D0) feasible, and (vi) patient or next of kin who has been informed of the terms of the study and has agreed to participate. Pre-existing immunosuppression was defined by the presence of solid or hematologic cancer, and/or chronic immunosuppressive treatment, and/or presence of innate or acquired immune deficiencies. Additional inclusion criteria for this ancillary study included an ICU length of stay of at least 10 days and a minimum of 7 days of dexamethasone treatment. Exclusion criteria were pregnancy, institutionalized patients, inability to obtain informed consent.

### Patient characteristics

For each patient, demographics, comorbidities, time from onset of COVID-19 symptoms to ICU admission, initial presentation of the disease in ICU including the ratio of the arterial partial pressure of oxygen to the fractional inspired oxygen (PaO2/FiO2 ratio) at admission and organ support during ICU stay were documented. Organ dysfunctions according to Sequential Organ Failure Assessment (SOFA) score (range 0–24, with higher scores indicating more severe organ failures), and Simplified Acute Physiology Score II (SAPS II; range, 0–164, with higher scores indicating greater severity of illness) were documented. Acute respiratory distress syndrome (ARDS) was defined if patients were invasively ventilated and met the Berlin criteria for ARDS [9]. Day 0 was considered as the day of inclusion in the clinical study (i.e. within 24h after ICU admission). Follow-up included ICU length of stay, in-hospital mortality, day-28 (D28) mortality, day-90 (D90) mortality, as well as occurrence of secondary infections using standardized diagnostic criteria based on French guidelines [10].

### Blood samples

Ethylene diamine tetra-acetic acid (EDTA-) anticoagulated blood was collected five times during the first month after ICU admission: within the first 48h after admission (Day 0: D0), between 72 and 96h after admission (D3), between D7 and D9 (D7), between D12 and D15 (D12), between D20 and D25 (D20). Blood was stored at 4–8 °C and processed within 4 h after withdrawal.

### Flow cytometry

CD4 + T lymphocyte subpopulation immunophenotyping was performed on an automated volumetric flow cytometer from Beckman Coulter (Aquios CL) as previously described [11]. The expression of monocyte HLA-DR (mHLA-DR) was determined using the Anti-HLA-DR/Anti-Monocyte Quantibrite assay (BD Biosciences, San Jose, USA). Total number of antibodies bound per cell (AB/C) were quantified using calibration with a standard curve determined with BD Quantibrite phycoerythrin (PE) beads (BD Biosciences) as described elsewhere [12]. Data were acquired on a Navios flow cytometer (Beckman Coulter, Hialeah, FL) and analyzed using Navios software (Beckman Coulter). Enumeration of lymphocyte subpopulations as well as mHLA-DR measurement were performed using standardized protocols fulfilling clinical and diagnostic laboratories accreditation requirements from the International Organization for Standardization.

### Statistical analysis

#### Descriptive statistics

Patients’ characteristics at ICU admission (i.e., demographics, comorbidities, COVID-19 symptoms, and organ support) and follow-up information were described using absolute (numbers) and relative (percentages) frequencies for qualitative variables, and median and interquartile range (Q1-Q3) for quantitative variables. Survivors and non survivors groups were compared using the Pearson χ2 test or Fisher exact test, as appropriate, for categorical variables and the Mann–Whitney U-test for continuous variables.

#### mHLA-DR and CD4 + T cell count trajectories modelling

The longitudinal evolution of mHLA-DR and CD4 + T lymphocyte count was modelled using linear mixed-effects models. In these models, mHLA-DR and CD4 + cell count trajectories were allowed to vary randomly and to deviate from the group average according to within- and between-individual variances. This method also adjusted for the within-subject correlation of the repeated observations over time, and for the inclusion of patients with a varying number of measurements. A second-order polynomial was used to model the effect of time entered as a continuous variable. This allowed to capture the decrease followed by an increase for mHLA-DR, and the increase followed by a plateau for CD4 + T cells during follow-up. The models were used to obtain predictions of mHLA-DR and CD4 + T lymphocyte count at day 10 for each patient.

#### Cut-off value calculations for discriminating Day-28 survivors and non survivors.

The best cut-off values of mHLA-DR and CD4 + T cell count at day 10 discriminating Day-28 survivors and non survivors patients were estimated by maximizing the Youden index and described by the area under the curve (AUC) of the corresponding receiver operating characteristics (ROC) curves.

#### Survival model

Day-28 survival estimates were calculated with the Kaplan–Meier product-limit method, and survival distributions were compared using the log-rank test. Day 0 was the day of ICU admission and patients remained included in the risk set until death or discharge. For the latter event, patients were considered as censored and were excluded of the risk set.

Univariate and multivariate Cox regressions were used to identify the variables associated with the risk of death before Day 28 and assessed by crude hazard ratio (HR) and adjusted HR (aHR) with their 95% confidence intervals (95%CI). Variables with a p value ≤ 0.20 in univariate analysis were entered in the multivariate models. Predictors included demographics, comorbidities, COVID-19 symptoms, and organ support at admission, as well as combinations of mHLA-DR and CD4 + T cell count at day 10.

In addition, mortality at day-90 was assessed by univariate and multivariate logistic regression analysis and assessed by crude odds ratio (OR) and adjusted OR (aOR). As previously, only variables with a p value ≤ 0.20 in univariate analysis were entered in the multivariate models.

P-values < 0.05 were considered as statistically significant. Analyses were performed using R version 4.0.3 [13].

## Results

### Description of the cohort

**Fig. 1:**
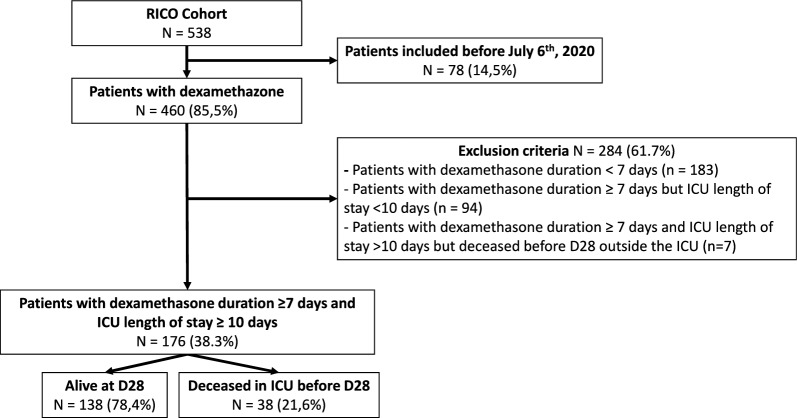
Study flow chart Patients included in the RICO clinical study before July 6th, 2020 (i.e. publication date of the RECOVERY clinical trial) were first excluded. Then exclusion criteria included a dexamethasone treatment duration below 7 days and an ICU length of stay below 10 days. Patients with dexamethasone treatment of at least 7 days and ICU length of stay of at least 10 days discharged alive from the ICU but who died with 28 days were excluded. Finally the cohort was divided between patients who were still alive at D28 and those who died in the ICU within 28 days after admission

From March 2020 to May 2022, the RICO study included 538 critically ill COVID-19 patients (See Table S1 and Figure S1 in the Online Data Supplement). Out of these, a total of 176 individuals stayed at least 10 days in the ICU and received dexamethasone (Fig. 1). Dexamethasone treatment was initiated upon ICU admission in 88% of these patients and was maintained for a median of 10 [9, 10] days. Table 1 depicts their clinical characteristics. Non-survivor patients at D28 were significantly older, presented with more comorbidities and required more frequently renal replacement therapy during the ICU stay compared to survivors (Table 1).

**Table 1:** Clinical characteristics of included patients according to their vital status at day 28

	All patients (n = 176)	Non-Survivors (n = 38)	Survivors (n = 138)	p-value
Demographics				
Age	67 [60—73]	71 [65—76]	66 [58—72]	**0.001**
Gender				> 0.05
Female	44 (25%)	6 (16%)	38 (28%)	
Male	123 (75%)	32 (84%)	100 (72%)	
Body mass index (kg/m^2^)	29.1 [26.2—33.6]	28.9 [25.8—32.6]	29.4 [26.3—34.0]	> 0.05
Body mass index ≥ 30 kg/m^2^ (%)				> 0.05
< 30	97 (56%)	22 (61%)	75 (55%)	
≥ 30	77 (44%)	14 (39%)	63 (45%)	
Missing	2	2	0	
Comorbidities				
Number of comorbidities				**0.015**
0	72 (41%)	9 (24%)	63 (46%)	
≥ 1	104 (59%)	29 (76%)	75 (54%)	
Charlson score	1.00 [0.00—2.00]	1.50 [1.00—3.00]	1.00 [0.00—1.00]	** < 0.001**
Pre-Existing immunosuppression				**0.022**
No	152 (86%)	28 (74%)	124 (90%)	
Yes, with chronic immunosuppressive therapy	21 (12%)	8 (21%)	13 (9.4%)	
Yes, without chronic immunosuppressive therapy	3 (1.7%)	2 (5.3%)	1 (0.7%)	
Symptoms at ICU Admission				
Delay between first symptoms (Days)	8 [6–10]	8 [5–9]	8 [6–10]	> 0.05
Missing	6	1	5	
Severity scores on ICU admission				
SOFA score	3 [1–6]	3 [1–5]	3 [1–6]	> 0.05
SAPS II score	32 [25—41]	33 [28—43]	32 [24—40]	> 0.05
PaO_2_/FiO_2_	94 [70—139]	100 [60—133]	92 [72—140]	> 0.05
Organ support on admission				
Invasive ventilation	44 (25%)	8 (21%)	36 (26%)	> 0.05
High flow nasal oxygen therapy	117 (67%)	28 (74%)	89 (65%)	
Standard oxygen therapy	10 (5.7%)	1 (2.6%)	9 (6.6%)	
Non-invasive ventilation	3 (1.7%)	1 (2.6%)	2 (1.5%)	
Vasopressor drugs				> 0.05
Yes	33 (19%)	6 (16%)	27 (20%)	
No	143 (81%)	32 (84%)	111 (80%)	
Organ support during ICU stay				
Renal replacement therapy				**0.005**
Yes	29 (16%)	12 (32%)	17 (12%)	
No	147 (84%)	26 (68%)	121 (88%)	
Invasive and non-invasive mechanical ventilation				**0.004**
Yes	130 (74%)	35 (92%)	95 (69%)	
No	46 (26%)	3 (7.9%)	43 (31%)	
Mechanical ventilation duration (days)	18 [11—30]	14 [10–17]	24 [13—37]	** < 0.001**
Follow-up				
Inclusion period				> 0.05
Covid wave 2: from July 6th 2020 till January 3rd 2021	62 (35%)	16 (42%)	46 (33%)	
Covid wave 3: from January 4th 2021 till July 4th 2021	72 (41%)	12 (32%)	60 (43%)	
Covid wave 4: after July 5th 2021	42 (24%)	10 (26%)	32 (23%)	
ICU length of stay (days)	22 [14—36]	17 [12–22]	27 [14—40]	** < 0.001**
Hospital length of stay (days)	33 [22—49]	19 [14–23]	40 [28—54]	** < 0.001**
Hospital mortality	56 (32%)	38 (100%)	18 (13%)	** < 0.001**
Day-90 mortality	54 (33%)	38 (100%)	16 (13%)	** < 0.001**
ICU acquired infections				> 0.05
Yes	97 (55%)	24 (63%)	73 (53%)	
No	79 (45%)	14 (37%)	65 (47%)	
ICU-acquired pneumopathy (% of ICU-acquired infections)	85 (88%)	22 (92%)	63 (86%)	> 0.05

Results are shown as medians and interquartile ranges [Q1-Q3] for continuous variables or numbers and percentages for categorical variables. Patients were separated in two groups based on their survival status at D28 after admission in intensive care unit (ICU). Sepsis-related organ failure assessment (SOFA) and Simplified acute physiology score II (SAPS II) scores were calculated during the first 24 h after ICU admission. Data between survivors and non-survivors were compared using non-parametric Mann–Whitney test for continuous variables or Fisher’s exact test for categorical variables. The number of missing values is indicated when necessary. In such case, percentages were calculated based on the total of available values. COVID-19 waves were defined based on data from Santé Publique France

**Fig. 2:**
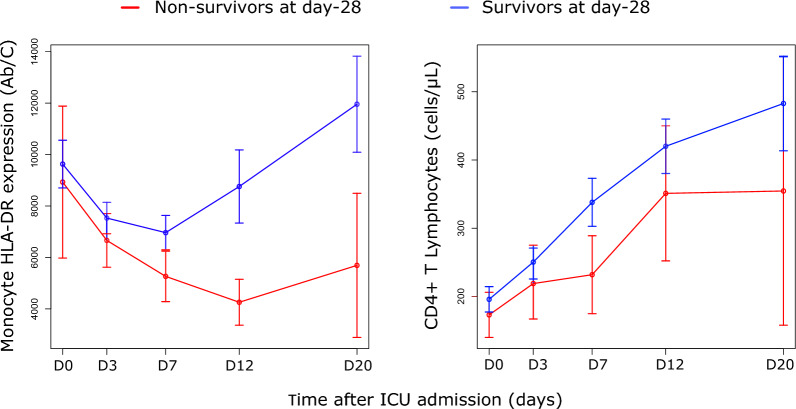
Kinetics of mHLA-DR and CD4 + T cell counts measured in D28 Survivors vs Non-Survivors critically ill COVID-19 patients. Values of monocyte HLA-DR expression (expressed as numbers of antibody bound per cell: AB/C, reference values: 13 500–45 000 AB/C) and CD4 + T cell absolute count (expressed as numbers of cells per µL, reference values: 365–1 345 cells/µL) measured in non-survivors (n = 38, red lines) and survivors (n = 138, blue lines) over time are shown. Patients were sampled within the first 48h after ICU admission (Day 0: D0), between 72 and 96h (D3), between D7 and D9 (D7), between D12 and D15 (D12), between D20 and D25 (D20). Results are presented as means and interquartile ranges (Q1-Q3). The numbers of available values for mHLA-DR and CD4 + count were 37 at D0 and at D3, 35 at D7, 27 at D12 and 10 at D20 in non-survivors. The numbers of available values were 132 for mHLA-DR and 134 for CD4 at D0, 130 for mHLA-DR and 128 for CD4 + at D3, 127 at D7, 102 for mHLA-DR and 103 for CD4 at D12 and 74 at D20 in survivors

Upon admission and till D7, both D28 survivors and non-survivors displayed decreased expression of monocyte HLA-DR (mHLA-DR) in comparison with lowest reference value from the lab (i.e., < 13500 AB/C, Fig. 2, Table S2) [14]. After day 7, D28 survivors experienced a sharp increase in mHLA-DR levels, while non-survivors remained at very low values (day 20 median < 5000 AB/C). Regarding CD4^+^ T cell count, both survivors and non-survivors initially had reduced CD4^+^ T lymphocyte values (median < 200 cells / μL, Fig. 2, Table 1). Over the course of monitoring, both groups gradually increased their CD4^+^ T cell counts until day 28. Notably, median value of CD4^+^ counts in D28 survivors was consistently higher compared to non-survivors throughout the entire monitoring period. That being said, at D20, 36% of D28 survivors and 70% of non-survivors presented with CD4^+^ T cell count below the lowest reference value from the lab (i.e. < 365 cells/µL [14]).

### Association between mHLA-DR at D10 and D28 mortality

We addressed whether mHLA-DR value and CD4^+^ T cell counts measured at the end of dexamethasone treatment were associated with D28 mortality. Since the initial study design did not include blood sampling at D10 (i.e. at the end of dexamethasone treatment) and to calculate both biomarkers’ values at this time point; we built mathematical models of biomarkers’ trajectories in critically ill COVID-19 patients. The global and individual modelling results are presented in Figures S2 and S3 in the Online Data Supplement, demonstrating the robustness of the models which were further used in association analyses.

**Fig. 3:**
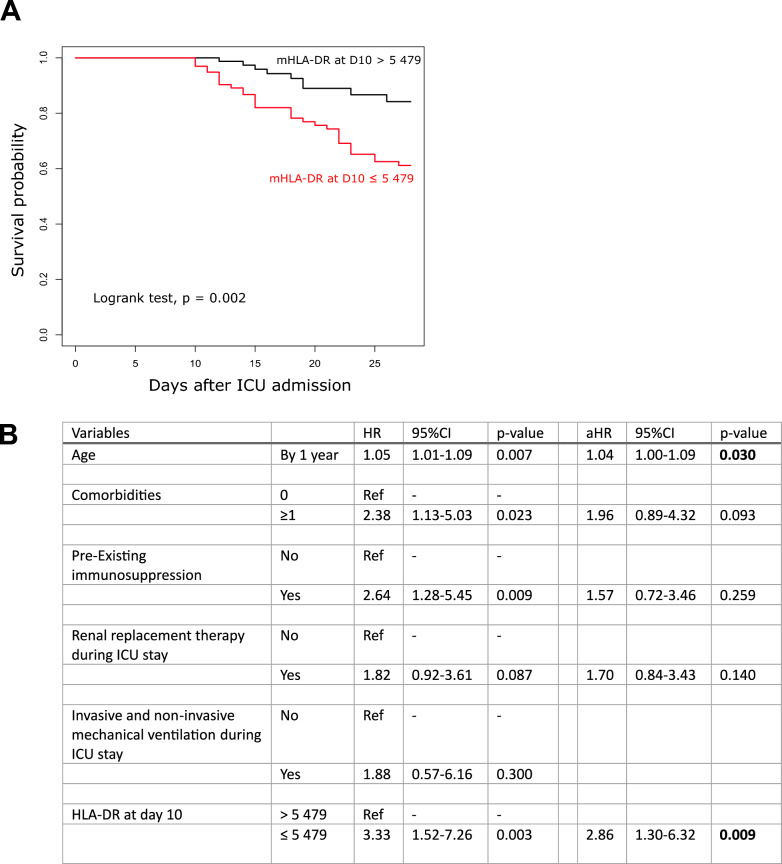
Association between mHLA-DR at D10 and mortality at D28 in critically ill COVID-19 patients. **A**. Based on cut-off value calculated using Youden index (i.e., 5 479 AB/C) from ROC curve analysis, patients were separated in 2 groups to build Kaplan–Meier survival curves. The Log-rank test was used to test the differences between these curves. **B**. Univariate and multivariate Cox regressions were used to identify the variables associated with the risk of death before day 28 and assessed by crude hazard ratio (HR) and adjusted HR (aHR) with their 95% confidence intervals (95%CI). Variables with a p value ≤ 0.20 in univariate analysis were entered in the multivariate models. mHLA-DR at day 10 in critically ill COVID-19 patients treated by dexamethasone was the predicted values from mathematical modeling of the change of this variable over time (n = 176)

At D10, we observed a significant association between low mHLA-DR levels and 28-day mortality in severe COVID-19 patients. The receiver operating characteristic (ROC) analysis for prediction of 28-day mortality based on mHLA-DR value at this time point yielded an area under the curve (AUC) value of 0.69 (95%CI 0.60–0.79, p < 0.001). After determining the best cut-off value using Youden index (i.e. 5,479 AB/C) on the ROC curve, Kaplan–Meier (KM) curves demonstrated substantial differences in survival between the groups defined by this threshold (Fig. 3A). This association remained significant in a multivariate analysis including usual clinical confounders such as age, presence of comorbidities or pre-existing immunosuppression (Fig. 3B). Of note, decreased mHLA-DR level at D10 was also independently associated with mortality at D90 (Tables S3 and S4).

### Association between CD4^+^ T cell count at D10 and D28 mortality

**Fig. 4:**
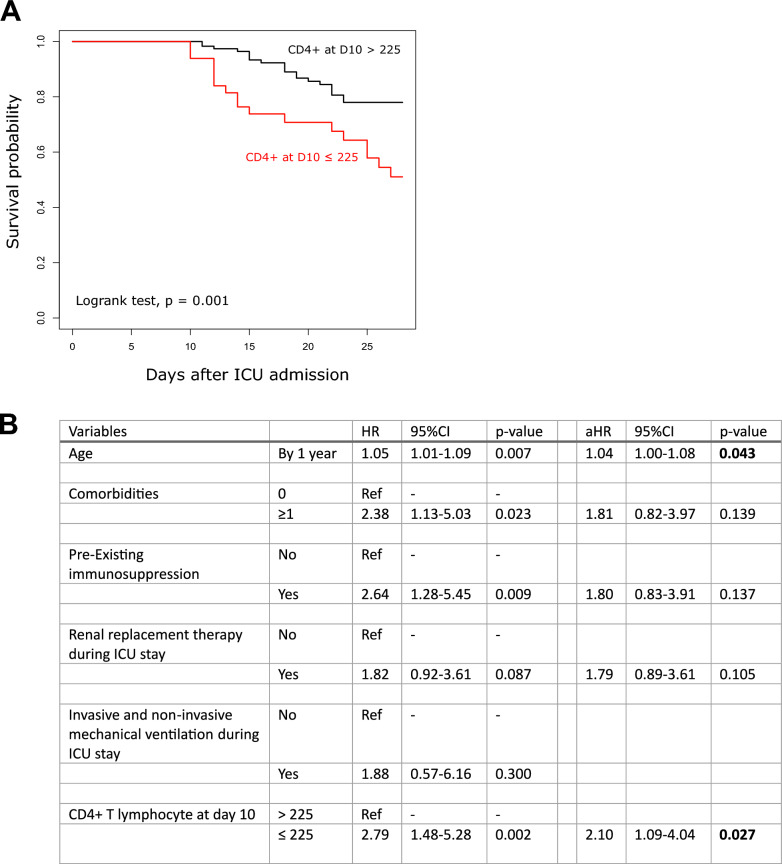
Association between CD4 + T lymphocyte count at D10 and mortality at D28 in critically ill COVID-19 patients. **A**. Based on cut-off value calculated using Youden index (i.e., 225 cells/µL) from ROC curve analysis, patients were separated in 2 groups to build Kaplan–Meier survival curves. Log-rank test was used to test the differences between these curves. **B**. Univariate and multivariate Cox regressions were used to identify the variables associated with the risk of death before Day 28 and assessed by crude hazard ratio (HR) and adjusted HR (aHR) with their 95% confidence intervals (95%CI). Variables with a p value ≤ 0.20 in univariate analysis were entered in the multivariate models. Number of circulating CD4 + T cells at day 10 in critically ill COVID-19 patients treated by dexamethasone was the predicted values from mathematical modeling of the change of this variable over time (n = 176)

Similarly, at D10, we observed a significant association between low CD4^+^ T cell count and mortality in severe COVID-19 patients. The receiver operating characteristic (ROC) analysis demonstrated an AUC of 0.63 (0.52–0.74, p < 0.001) for the prediction of D28 mortality based on CD4^+^ T cell count measured at D10. Upon determining the optimal cut-off value of 225 cells/μL with the ROC curve curve and Youden index, KM curves revealed statistically significant difference in survival between the two groups defined by this threshold (Fig. 4A). Importantly, this association remained significant even after conducting multivariate analysis including usual clinical confounders (Fig. 4B).

### Combination of mHLA-DR and CD4 + T cell count at D10 to predict D28 mortality

**Fig. 5:**
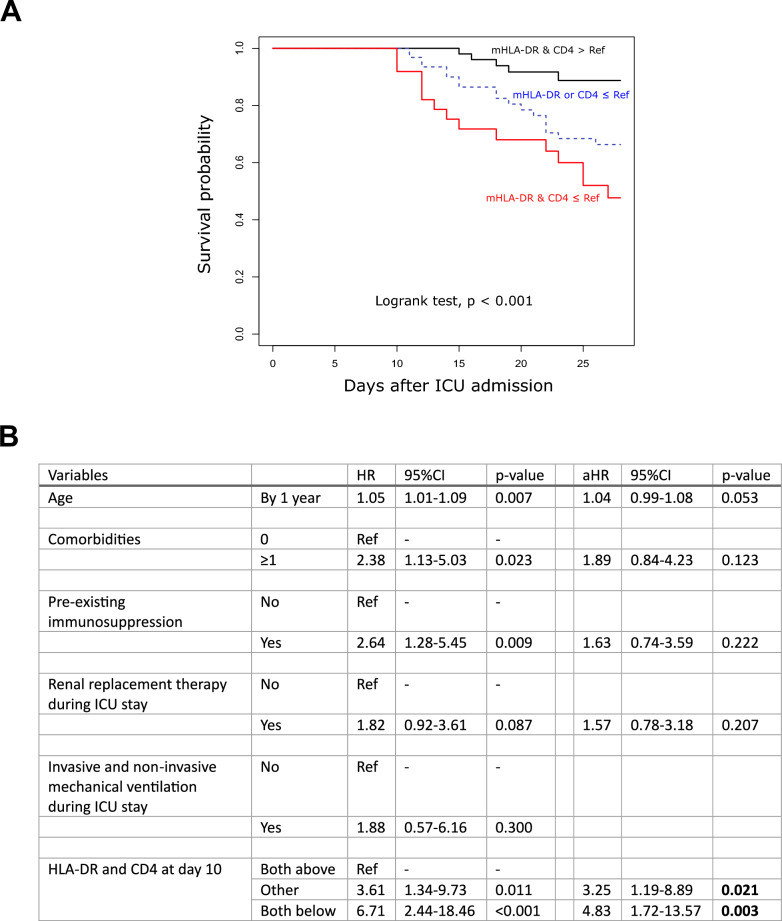
Association between the combination of mHLA-DR and CD4 + T lymphocyte count at D10 and mortality at D28 in critically ill COVID-19 patients. **A**. Patients were categorized into three groups based on mHLA-DR and CD4 + T cell count at day 10: Group 1 = both markers (mHLA-DR and CD4) above their respective cut-off values calculated using Youden index (i.e. 5 479 AB/C for mHLA-DR and 225 cells/µL for CD4 + T cell count) (black line); Group 2 = at least one marker (either mHLA-DR or CD4) above its cut-off value (red line); Group 3 = Both markers below their cut-off values (green line). Log-rank test was used to test the differences between these curves. **B**. Univariate and multivariate Cox regressions were used to identify the variables associated with the risk of death before Day 28 and assessed by crude hazard ratio (HR) and adjusted HR (aHR) with their 95% confidence intervals (95%CI). Variables with a p value ≤ 0.20 in univariate analysis were entered in the multivariate models. Monocyte HLA-DR expression and number of circulating CD4 + T cells at day 10 in critically ill COVID-19 patients treated by dexamethasone were calculated based mathematical modeling

Subsequently, we investigated whether the inclusion of both markers in the same analysis could provide additional clinical information. Patient were categorized into three groups based on mHLA-DR and CD4^+^ T cell count measured at D10: Group 1 = both markers (mHLA-DR and CD4) above their respective cut-off values as previously calculated; Group 2 = at least one marker (either mHLA-DR or CD4) above their cut-off value; Group 3 = both markers below or equal to their cut-off values. Patients included in Group 3 exhibited a significantly lower survival compared with other groups as observed on KM curves (Fig. 5A). When included in a multivariate analysis, being included in Group 3 was associated with a hazard ratio (HR) of 4.83 (1.72–13.57) regarding the risk of death at D28 after adjustment for other important prognostic co-variables (Fig. 5B). The mortality at D28 in this group exceeded 60%.

## Discussion

In this study focusing on critically ill COVID-19 patients undergoing dexamethasone treatment, we examined mHLA-DR values and CD4 + T cell counts over time. After completion of a 10-day dexamethasone regimen, we showed that these immune parameters can distinguish a subgroup of patients with high mortality risk.

During the COVID-19 pandemic, the introduction and utilization of dexamethasone marked a significant milestone in the treatment of severe cases [4, 5]. As it was discovered that the virus could trigger an exaggerated immune response that caused severe damage to organs and tissues, particularly within the lungs [1], treatment with dexamethasone was assessed due to its potent anti-inflammatory properties. The landmark RECOVERY trial [2], conducted in the United Kingdom, demonstrated that dexamethasone reduced mortality rates in hospitalized COVID-19 patients requiring oxygen or ventilator support. Then, additional trials (*e.g*., REMAP-CAP Trial [3], CoDEX Trial [15]), also found that dexamethasone reduced the duration of mechanical ventilation and improved clinical outcomes in these patients. Overall, these trials, along with other observational studies, have provided evidence supporting the use of dexamethasone in the management of severe COVID-19 cases (*i.e.,* those who require supplemental oxygen or ventilator support). This led to the widespread and successful adoption of dexamethasone by healthcare providers around the world in the treatment of severe COVID-19 cases. Despite this progress, heterogeneity of ICU patients and of their immune phenotypes remain challenging in clinical decision-making once regular 10-day dexamethasone treatment is completed [6]. Taking this into account, a group of experts conceptualized in Nature Medicine in 2022 the principle of monitoring patients' immune functions at the end of dexamethasone treatment to guide subsequent immunomodulation approaches [6]. However, to the best of our knowledge, there has been no report on immune parameters in this specific context. Therefore, our results obtained from a substantial cohort of homogeneous patients, namely critically ill COVID-19 patients who spent a minimum of 10 days in the ICU and received a complete course of dexamethasone, offer highly informative and valuable insights as they demonstrate the diversity of patients’ immune profiles. This reinforces the relevance of biomarker-guided complementary immunomodulation approaches [6].

The first significant result from this study is the observation that after 10 days in the ICU, severe COVID-19 patients exhibit signs of immunosuppression. Indeed, despite a slight increase in the number of CD4^+^ T cells over time, the number of circulating lymphocytes remained very low at day 10. Similarly, regarding mHLA-DR, a constant decrease was observed over the first week after ICU admission. These data are consistent with prior literature findings, albeit acquired through diverse methodologies for assessing mHLA-DR expression, demonstrating immune dysregulation in COVID-19 patients across different severity levels. [1, 16–22]. However, the current data were obtained in a cohort of ICU COVID-19 patients, strictly focusing on those undergoing 10-day dexamethasone treatment and using a standardized method of mHLA-DR evaluation.

In addition, we could demonstrate that at day 10, patients with lower values of each marker were much more prone to die within the next 28 days. Indeed, both markers were significantly associated with one month and 3 month mortality independently of usual clinical risk factors. In particular, these results demonstrated a greater association for patients' outcomes compared to age and comorbidities, which are typically considered as confounding factors in forecasting COVID-19 mortality. Most importantly, when both markers were combined, they identified a phenotype that was also independently associated with unfavorable outcome, reaching extremely high mortality (> 60%). These association studies highlight the role of altered immune response in the prognosis of critically ill COVID-19 patients. As previously described in bacterial sepsis [7, 8, 23], in addition with other clinical parameters such as increased age, presence of co-morbidities or pre-existing immunosuppression, the incapacity of patients to appropriately regulate their immune response induced by an infectious trigger represents an additional and independent risk factor of mortality. Of note, these mHLA-DR and CD4^+^ values agree with other clinical conditions characterized by immunosuppression. Regarding CD4^+^ T cell count, for illustrative purposes, WHO guidelines define an advanced HIV disease based on a CD4^+^ T cell count below 200 cells/µL. In the context of septic shock, current clinical trials involving the use of IFN-γ to enhance immune functions are enrolling patients with mHLA-DR levels below 8,000 AB/C (Ignorant [NCT05843786], Immunosep [NCT04990232]) in accordance with the idea of moving away from a "one size fits all" approach towards a more individualized practice [6, 24]. As COVID-19 triggers a complex response characterized by the simultaneous or successive manifestation of pro-inflammatory and anti-inflammatory/immunosuppressive elements that disrupt the mechanisms intended to maintain homeostasis [1], it appears therefore appropriate to consider sequential immune-adjuvant therapeutics, potentially with opposing effects, tailored to each patient's needs in order to address the complex immunological dynamics seen in the disease [6].

This study does have limitations. The absence of a control group consisting of patients not treated with dexamethasone could be criticized. However, since our intention was not to question dexamethasone usage or to evaluate its putative anti-inflammatory impact on measured immune parameters, but rather to assess the immune profiles specifically at the conclusion of a 10-day dexamethasone course [6], we believe that such control group may not be necessary. Similarly, the assessment of potential dexamethasone impact on immune function in comparison with other therapeutic approaches in COVID-19 and in different ICU conditions could be the subject of further studies. Finally, the putative impact of vaccination on immune profile was not assessed in the present study.

In conclusion, our results underscore the importance of incorporating immune monitoring with CD4^+^ T cell count and mHLA-DR into the prognostic evaluation of severe COVID-19 patients upon a full dexamethasone treatment course. By utilizing standardized immunomonitoring tools available in clinical practice, it is conceivable to better track the patient's immune status evolution, thereby guiding subsequent immunomodulation options.

### Supplementary Information

Supplementary material 1.

Supplementary material 2.

## Data Availability

The datasets analyzed during the current study are available from the corresponding author on reasonable request.

## Abbreviations

AB/C: Numbers of antibodies bound per cell
ARDS: Acute respiratory distress syndrome
AUC: Area under the curve
CD4: CD4 + T lymphocyte
CI: Confidence interval
COVID-19: Coronavirus Disease 2019
EDTA: Ethylenediaminetetra-acetic acid
HLA-DR: Human leukocyte antigen-DR
HR: Hazard ratio
aHR: Adjusted HR
ICU: Intensive care unit
RICO: REA-IMMUNO-COVID
ROC: Receiver operating characteristic
RT-PCR: Reverse transcriptase polymerase chain reaction
SAPS II: Simplified Acute Physiology Score II
SOFA: Sequential Organ Failure Assessment score
PaO_2_/FiO_2_ ratio: Ratio of the arterial partial pressure of oxygen to the fractional inspired oxygen
