# Supplementary material for: Monitoring monocyte HLA-DR expression and CD4 + T lymphocyte count in dexamethasone-treated severe COVID-19 patients
Source: Ann Intensive Care. 2024 May 18;14:76. doi: 10.1186/s13613-024-01310-5 (PMC11102415; doi:10.1186/s13613-024-01310-5)
Supplement: Supplementary file 1 — Supplementary material 1. [file 13613_2024_1310_MOESM1_ESM.pdf]

Monitoring monocyte HLA-DR expression and CD4+ T lymphocyte count in dexamethasone-treated severe COVID-19 patients

Guillaume Monneret[1,2], Nicolas Voirin[3], Jean-Christophe Richard[4], Martin Cour[5], Thomas Rimmelé[2,6], Lorna Garnier[7], Hodane Yonis[4], Remy Coudereau[1,2], Morgane Gossez[1,8], Florent Wallet[9], Marie-Charlotte Delignette[10], Frederic Dailler[11], Marielle Buisson[12], Laurent Argaud[4], Anne-Claire Lukaszewicz[2,5], Fabienne Venet[1,8] for the RICO study group

ONLINE DATA SUPPLEMENT

<u>Table S1.</u> Clinical characteristics of critically ill patients with COVID-19 included in the RICO clinical study (n=538)

Results are shown as medians and interquartile ranges [Q1-Q3] for continuous variables or numbers and percentages for categorical variables. Sepsis-related organ failure assessment (SOFA) and Simplified acute physiology score II (SAPS II) scores were calculated during the first 24h after intensive care unit (ICU) admission. ARDS: acute respiratory distress syndrome. In case of missing values, the number of missing values is indicated. In such case, percentages were calculated based on the total of available values. COVID-19 waves were defined based on data from Santé Publique France.

	All patients (n = 538)
Demographics	
Age (years)	65 [56 – 72]
Gender<br>Female<br>Male	 155 (29%)<br>383 (71%)
Body mass index (kg/m^2)	28.7 [25.8 - 32.7]
Missing	6
Body mass index ≥ 30 kg/m^2 (%)	211 (40%)
Missing	6
Comorbidities	
Number of comorbidities	
0	247 (46%)
≥ 1	291 (54%)
Charlson score	1 [0 – 2]
Pre-existing Immunosuppression	
No	489 (91%)
Yes, with chronic immunosuppressive therapy	43 (8.0%)
Yes, without chronic immunosuppressive therapy	6 (1%)
Admission symptoms	
Delay between first symptoms (Days)	9 [6 – 11]
Missing	10
ARDS on ICU admission (%)	
Yes	128 (24%)
No	410 (76%)
Severity scores on ICU admission	
SOFA score	3.0 [1.0 - 5.0]
SAPS II score	31 [24 – 40]
PaO_2/FiO_2	105 [78 – 161]
Organ support on ICU admission	
Ventilation<br>Invasive mechanical ventilation<br>High flow nasal oxygen therapy<br>Standard oxygen therapy<br>Non-invasive ventilation<br>No oxygen support<br>Missing	 104 (20%)<br>350 (66%)<br>66 (12%)<br>11 (2.1%)<br>2 (0.4%)<br>5
Vasoactive drugs	77 (14%)
Organ support during ICU stay	
Renal replacement therapy	
Yes	54 (10%)

	All patients (n = 538)
No	483 (90%)
Invasive and non-invasive mechanical ventilation	
Yes	235 (44%)
No	303 (56%)
Invasive and non-invasive mechanical ventilation duration (days)	16 [7 – 29]
Follow-up	
Inclusion period	
Covid wave 1: from March 2nd 2020 till July 5th 2020	67 (12%)
Covid wave 2: from July 6th 2020 till January 3rd 2021	182 (34%)
Covid wave 3: from January 4th 2021 till July 4th 2021	176 (33%)
Covid wave 4: after July 5th 2021	113 (21%)
Days in ICU length of stay (days)	9 [5 – 20]
Days in Hospital length of stay (days)	18 [11 – 34]
Hospital mortality	123 (23%)
Day-28 mortality	94 (18%)
Day-90 mortality	121 (26%)
ICU acquired infections	
Yes	167 (31%)
No	371 (69%)
ICU-acquired pneumopathy (% of ICU-acquired infections)	158 (95%)

Table S2. mHLA-DR and absolute CD4+ T cell count monitoring during 20-day follow-up in whole cohort, 28-day survivors and non survivors

Monocyte HLA-DR expression and absolute count of CD4+ T cells were monitored in critically ill COVID-19 patients (n = 176 patients). Measured values at Day 0 (i.e. within the first 48h after admission), Day 3 (between 72h and 96h after admission), Day 7 (between D7 and D9), Day 12 (between D12 and D15), Day 20 (between D20 and D25) are shown. In addition, values calculated by the models at Day 10 are presented (* marked by an asterisk). Results in survivors (n = 138) and non-survivors (n = 38) at day 28 are presented. Percentages of patients with mHLA-DR or CD4+ T cell count below lowest reference value for each marker are shown. Results are expressed as medians and interquartile ranges [Q1-Q3]. Monocyte HLA-DR is presented as numbers of antibodies bound per cells (AB/C, reference values: 13 500 – 45 000 AB/C). Circulating number of CD4+ T cells is expressed as numbers of cells per µl (reference values: 365 – 1 345 cells/µL).

**Whole cohort (n = 176)**	**Day 0**	**Day 3**	**Day 7**	**Day 10***	**Day 12**	**Day 20**
mHLA-DR (AB/C)	8,116 [5,706 - 11,436]	6,658 [4,718 - 9,140]	5,693 [4,008 - 7,950]	5,509 [4,135 - 7,726]	5,737 [3,443 - 9,681]	8,436 [4,960 - 16,212]
% of patients below normal values	86	95	94	98	86	69
CD4 T (cells / µL)	168 [105 – 248]	205 [125 – 338]	279 [160 – 400]	329 [205 – 449]	389 [230 – 547]	400 [245 – 645]
% of patients below normal values	93	79	69	59	43	40
**Survivors (n = 138)**						
mHLA-DR (AB/C)	9,078 [5,724 - 11,620]	6,793 [5,071 - 9,436]	5,932 [4,225 - 8,268]	5,942 [4,423 - 8,007]	6,183 [3,763 - 11,125]	9,201 [5,966 - 17,138]
% of patients below normal values	85	95	92	98	82	65
CD4 T (cells/ µL)	170 [110 – 249]	210 [140 – 338]	299 [194 – 434]	341 [235 – 463]	408 [280 – 554]	403 [260 – 658]
% of patients below normal values	93	80	65	56	40	36
**Non-Survivors (n = 38)**						
mHLA-DR (AB/C)	7,152 [5,644 - 8,823]	5,846 [4,414 - 8,730]	4,818 [3,030 - 7,020]	4,303 [3,673 - 5,232]	3,571 [2,838 - 4,982]	4,804 [3,732 - 6,045]
% of patients below normal values	92	95	100	100	100	100
CD4 T (cells / µL)	161 [95 – 216]	161 [102 – 315]	165 [111 – 348]	241 [131 – 389]	266 [124 – 523]	249 [154 – 412]
% of patients below normal values	95	76	80	68	56	70

Table S3. Clinical characteristics of critically ill patients with COVID-19 according to vital status at day 90 (n=163).

Out of 176 patients, information about vital status at day-90 was available in 163, while 13 patients had missing data. Results are shown as medians and interquartile ranges [Q1-Q3] for continuous variables or numbers and percentages for categorical variables. Patients were separated in two groups based on survival status at D90. Sepsis-related organ failure assessment (SOFA) and Simplified acute physiology score II (SAPS II) scores were calculated during the first 24h after intensive care unit (ICU) admission. Data between survivors and non-survivors were compared using non-parametric Mann-Whitney test for continuous variables or Fisher's exact test for categorical variables. ARDS: acute respiratory distress syndrome. In case of missing values, the number of missing values is indicated. In such case, percentages were calculated based on the total of available values. COVID-19 waves were defined based on data from Santé Publique France.

	All patients (n = 163)	Non-Survivors (n = 54)	Survivors (n = 109)	p-value
Demographics				
Age	67 [60 – 73]	71 [64 – 75]	66 [58 – 71]	**< 0.001**
Gender				**0.004**
Female	41 (25%)	6 (11%)	35 (32%)	
Male	122 (75%)	48 (89%)	74 (68%)	
Body mass index (kg/m^2)	29.4 [26.3 - 33.8]	27.9 [25.2 - 32.6]	30.0 [26.6 - 34.4]	**0.049**
Missing	2	2	0	
Body mass index ≥ 30 kg/m^2 (%)				> 0.05
< 30	88 (55%)	33 (63%)	55 (50%)	
≥ 30	73 (45%)	19 (37%)	54 (50%)	
Missing	2	2	0	
Comorbidities				
Comorbidities				> 0.05
0	65 (40%)	17 (31%)	48 (44%)	
≥ 1	98 (60%)	37 (69%)	61 (56%)	
Charlson score	1.00 [0.00 - 2.00]	1.00 [0.00 - 3.00]	1.00 [0.00 - 1.00]	**0.027**
Pre-existing immunosuppression				> 0.05
No	139 (85%)	44 (81%)	95 (87%)	
Yes, with chronic immunosuppressive therapy	21 (13%)	8 (15%)	13 (12%)	
Yes, without chronic immunosuppressive therapy	3 (1.8%)	2 (3.7%)	1 (0.9%)	
ICU Admission symptoms				
Delay between first symptoms (Days)	8.0 [6.0 - 10.0]	8.0 [6.0 - 9.0]	8.0 [6.0 - 10.0]	> 0.05
Missing	4	1	4	
ARDS on ICU admission (%)				> 0.05
Yes	47 (29%)	15 (28%)	32 (29%)	
No	116 (71%)	39 (72%)	77 (71%)	
Severity scores on ICU admission				
SOFA score	3.0 [1.0 - 6.0]	3.0 [1.0 - 6.0]	2.0 [1.0 - 6.0]	> 0.05
SAPS II score	32 [25 – 42]	34 [29 – 43]	31 [24 – 38]	> 0.05
PaO_2/FiO_2	92 [69 – 140]	95 [66 – 130]	91 [72 – 148]	> 0.05
Organ support on ICU admission				
Invasive mechanical ventilation	42 (26%)	13 (24%)	29 (27%)	> 0.05

	All patients (n = 163)	Non-Survivors (n = 54)	Survivors (n = 109)	p-value
High flow nasal oxygen therapy<br>Standard oxygen therapy<br>Non-invasive ventilation<br>Missing values	107 (66%)<br>9 (5.6%)<br>3 (1.9%)<br>2	39 (72%)<br>1 (1.9%)<br>1 (1.9%)<br>0	68 (64%)<br>8 (7.5%)<br>2 (1.9%)<br>2	
Vasoactive drugs				> 0.05
Yes	31 (19%)	9 (17%)	22 (20%)	
No	143 (81%)	32 (84%)	111 (80%)	
Organ support during ICU stay				
Renal replacement therapy				**< 0.001**
Yes	29 (18%)	20 (37%)	9 (8.3%)	
No	134 (82%)	34 (63%)	100 (92%)	
Invasive and non-invasive mechanical ventilation				**< 0.001**
Yes	123 (75%)	51 (94%)	72 (66%)	
No	40 (25%)	3 (5.6%)	37 (34%)	
Invasive and non-invasive Mechanical ventilation duration (days)	13 [0 – 27]	16 [10 – 25]	11 [0 – 27]	> 0.05
Follow-up				
Inclusion period				> 0.05
Covid wave 2: from July 6th 2020 till January 3rd 2021	60 (37%)	21 (39%)	39 (36%)	
Covid wave 3: from January 4th 2021 till July 4th 2021	70 (43%)	19 (35%)	51 (47%)	
Covid wave 4: after July 5th 2021	33 (20%)	14 (26%)	19 (17%)	
Days in ICU	21 [14 – 37]	20 [14 – 29]	22 [13 – 40]	> 0.05
Days in Hospital	34 [22 – 51]	22 [16 – 30]	41 [27 – 58]	**< 0.001**
Hospital mortality	56 (35%)	54 (100%)	2 (1.9%)	**< 0.001**
ICU acquired infections				**0.048**
Yes	94 (58%)	37 (69%)	57 (52%)	
No	69 (42%)	17 (31%)	52 (48%)	
ICU-acquired pneumopathy (% of ICU-acquired infections)	82 (87%)	33 (89%)	49 (86%)	> 0.05

<u>Table S4</u>. Univariate and multivariate analysis evaluating the association between mHLA-DR and CD4+ T cell count at day 10 and mortality at day 90 (n=163).

Logistic regression analyses were used to identify the variables associated with the risk of death before Day 90 and assessed by odds ratio (OR) and adjusted OR (aOR) with their 95% confidence intervals (95%CI). Variables with a p value ≤ 0.20 in univariate analysis were entered in the multivariate models.

Variables		OR	95%CI	p-value		aOR	95%CI	**p-value**
Age	By 1 year	1.06	1.02-1.10	**0.001**		1.10	10.04-1.16	**< 0.001**
Gender	Female	Ref	-	-		Ref	-	-
	Male	3.66	1.44-9.30	**0.006**		3.74	1.18-11.83	**0.025**
BMI	By 1 unit	0.98	0.94-1.02	> 0.05				
Charlson score	By 1 unit	1.25	1.02-1.52	**0.028**		1.26	0.95-1.66	> 0.05
Renal replacement therapy at day 10	No	Ref	-	-		Ref	-	-
	Yes	7.32	3.05-17.57	**< 0.001**		3.55	1.24-10.14	**0.018**
Invasive and non-invasive mechanical ventilation at day 10	No	Ref	-	-		Ref	-	-
	Yes	9.37	2.76-31.82	**< 0.001**		14.87	3.40-64.97	**< 0.001**
HLA-DR at day 10 (threshold = 5 479 AB/C)	Above	Ref	-	-		Ref	-	-
	Below	5.51	2.63-11.53	**< 0.001**		3.53	1.44-8.62	**0.006**
CD4 at day 10 (threshold = 225 cell / µL)	Above	Ref	-	-		Ref	-	-
	Below	3.07	1.53-6.15	**0.002**		1.42	0.57-3.54	> 0.05

<u>Figure S1</u>. Number of inclusions per week in the RICO clinical study according to treatment with dexamethasone.

Number of inclusions of patients under dexamethasone treatment are shown in gray bars. Inclusions of patients without dexamethasone treatment are shown in open bars. Inclusions started on week 11, 2020 and were stopped on week 18, 2022.

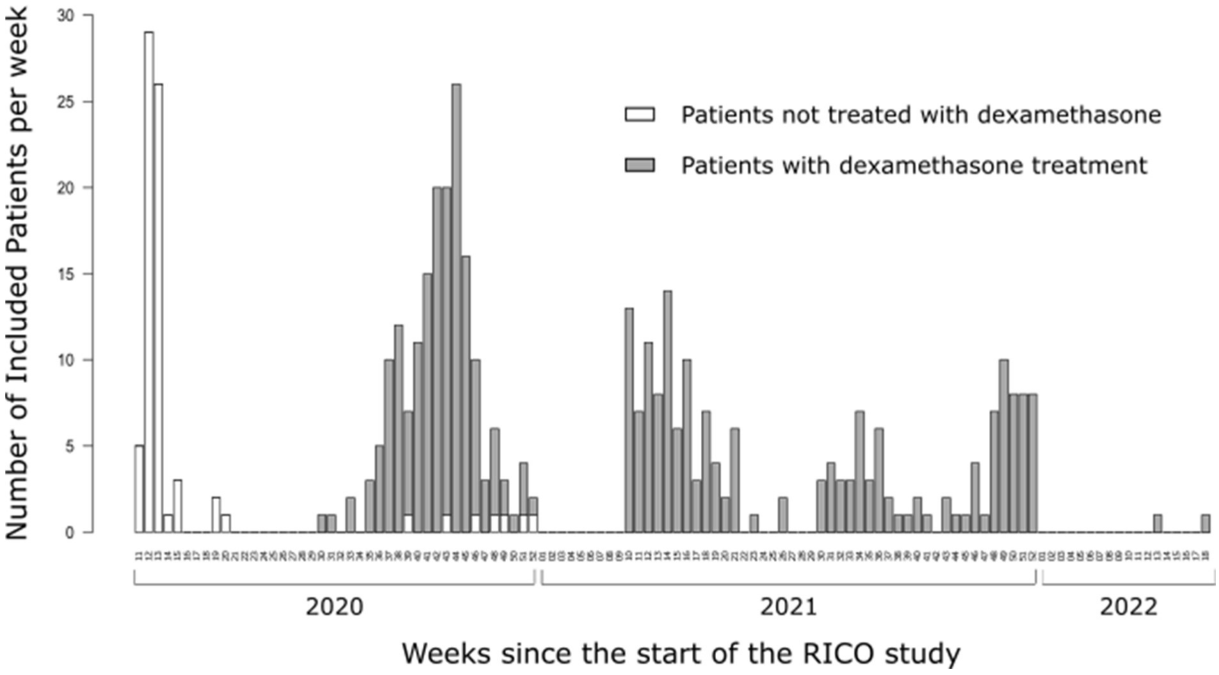

Figure S2. Modelization of mHLA-DR and CD4+ T cell count overtime in critically ill COVID-19 patients.

Red lines represents medians values of mHLA-DR and CD4+T cell counts as calculated by the models at the respective time-points. In regard, measured values obtained in the cohort are presented as grey box-plots.

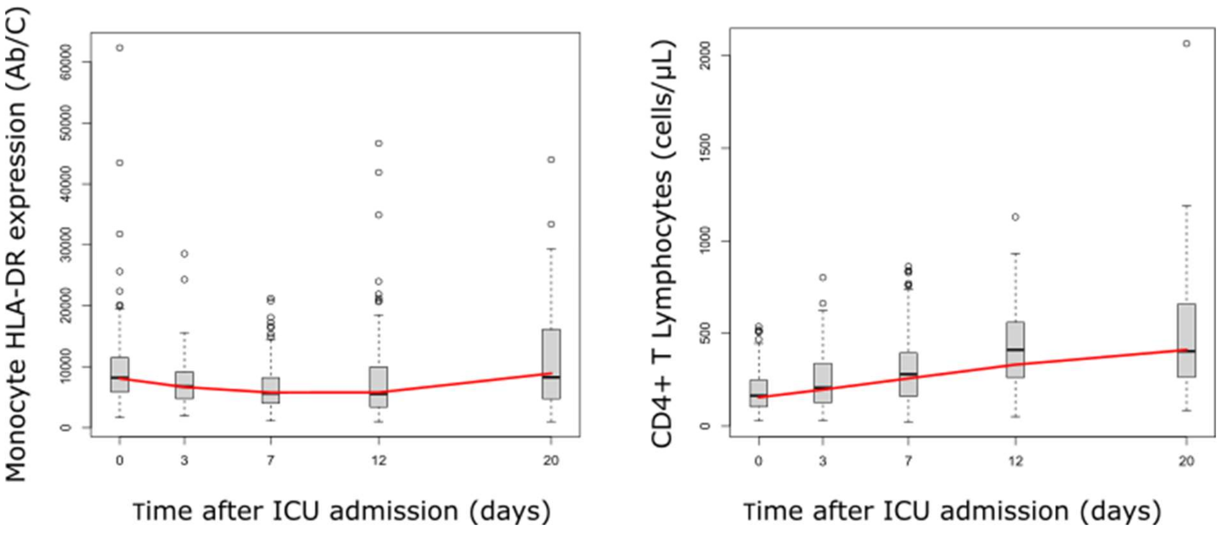

Figure S3. Modelization of mHLA-DR and CD4+ values overtime in critically ill COVID-19 patients: individual results.

Individual trajectories as calculated by the models vs measured values for mHLA-DR and CD4+ T cell count are shown for all the patients included in the cohort.

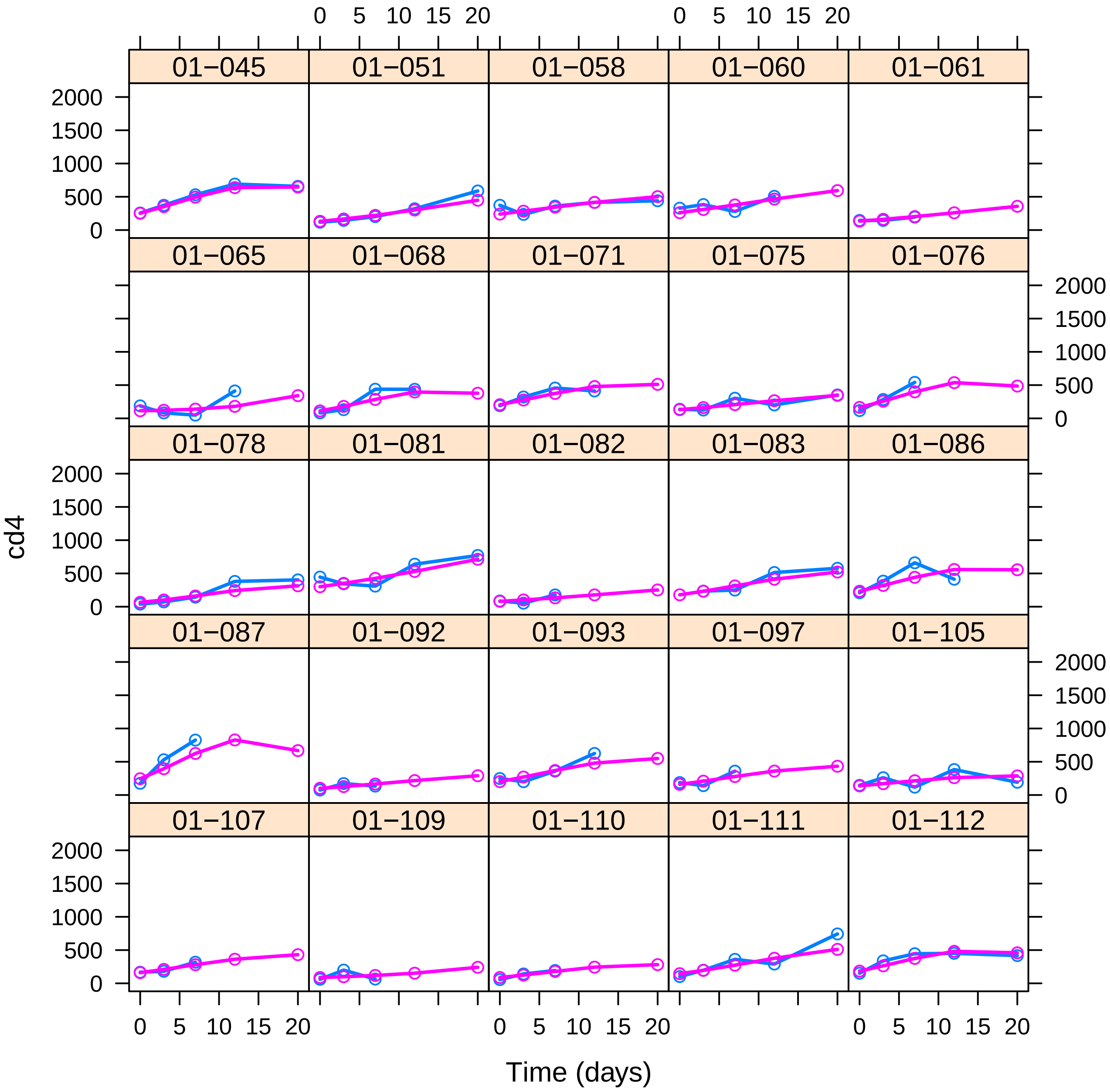
01−045
01−051
01−058
01−060
01−061
01−065
01−068
01−071
01−075
01−076
01−078
01−081
01−082
01−083
01−086
01−087
01−092
01−093
01−097
01−105
01−107
01−109
01−110
01−111
01−112
cd4
Time (days)
0 5 10 15 20
0 500 1000 1500 2000

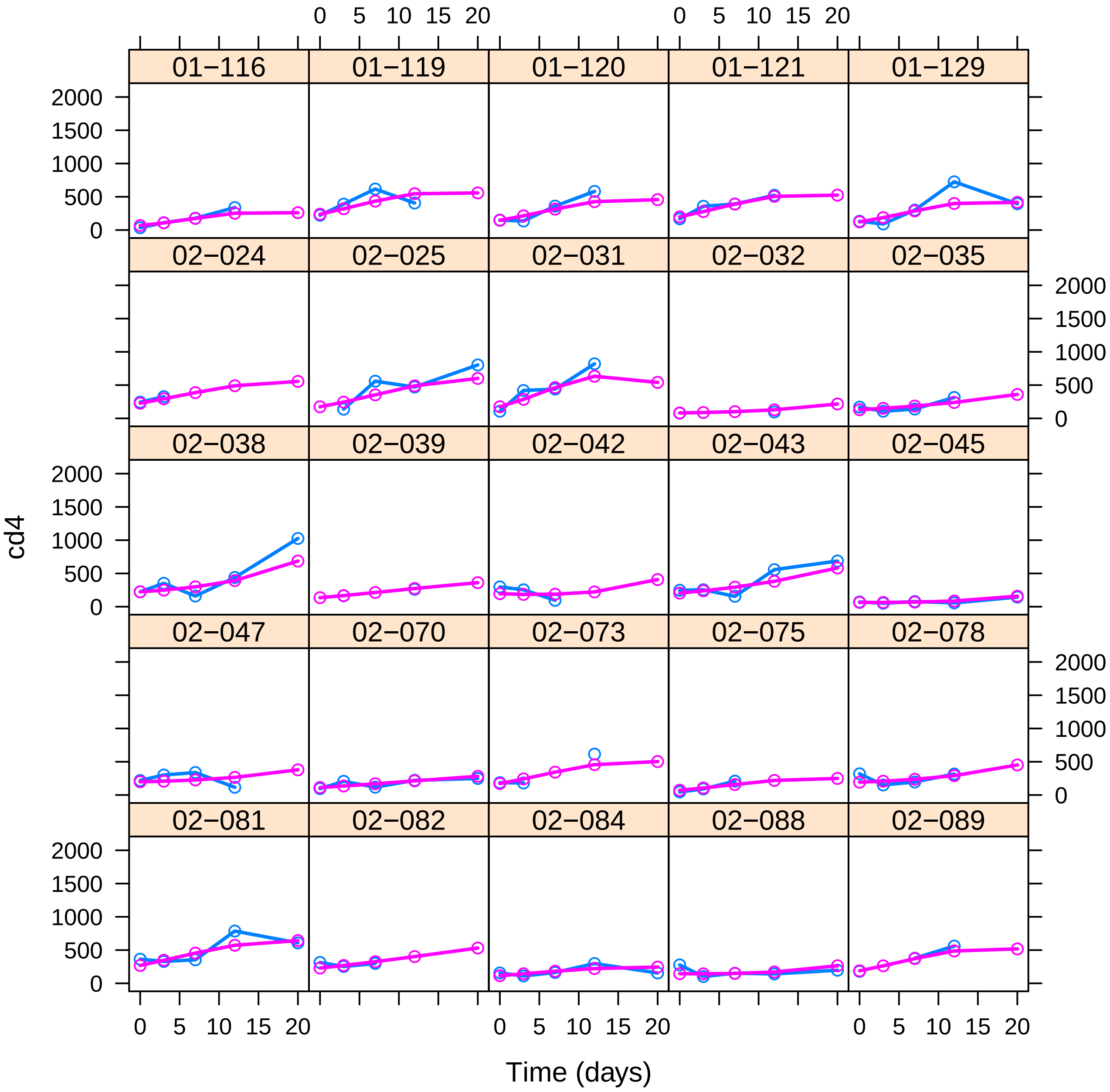
01−116
01−119
01−120
01−121
01−129
02−024
02−025
02−031
02−032
02−035
02−038
02−039
02−042
02−043
02−045
02−047
02−070
02−073
02−075
02−078
02−081
02−082
02−084
02−088
02−089
cd4
0
500
1000
1500
2000
0
5
10
15
20
Time (days)

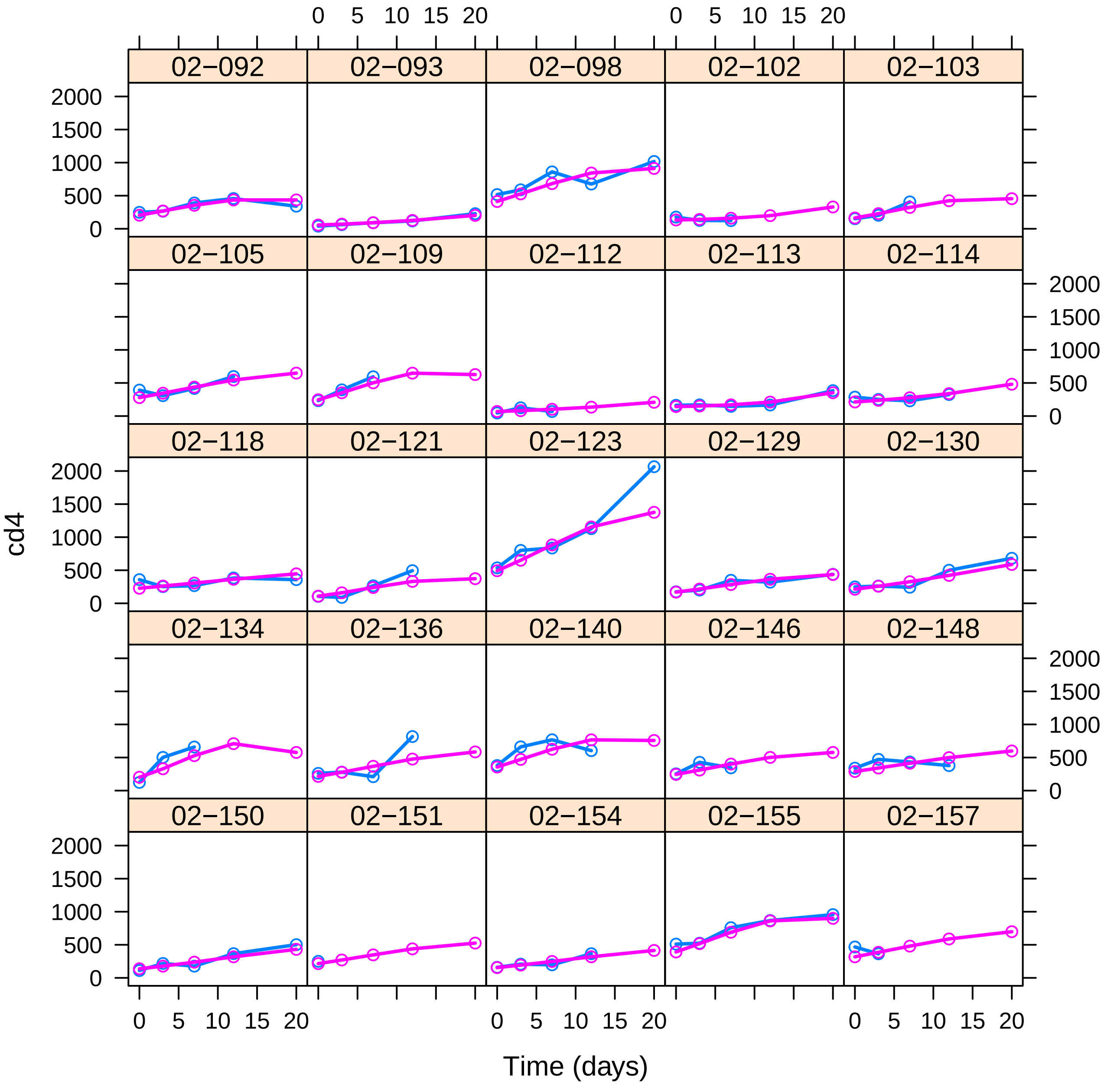

02−092
02−093
02−098
02−102
02−103
02−105
02−109
02−112
02−113
02−114
02−118
02−121
02−123
02−129
02−130
02−134
02−136
02−140
02−146
02−148
02−150
02−151
02−154
02−155
02−157
cd4
0
500
1000
1500
2000
0
5
10
15
20
Time (days)

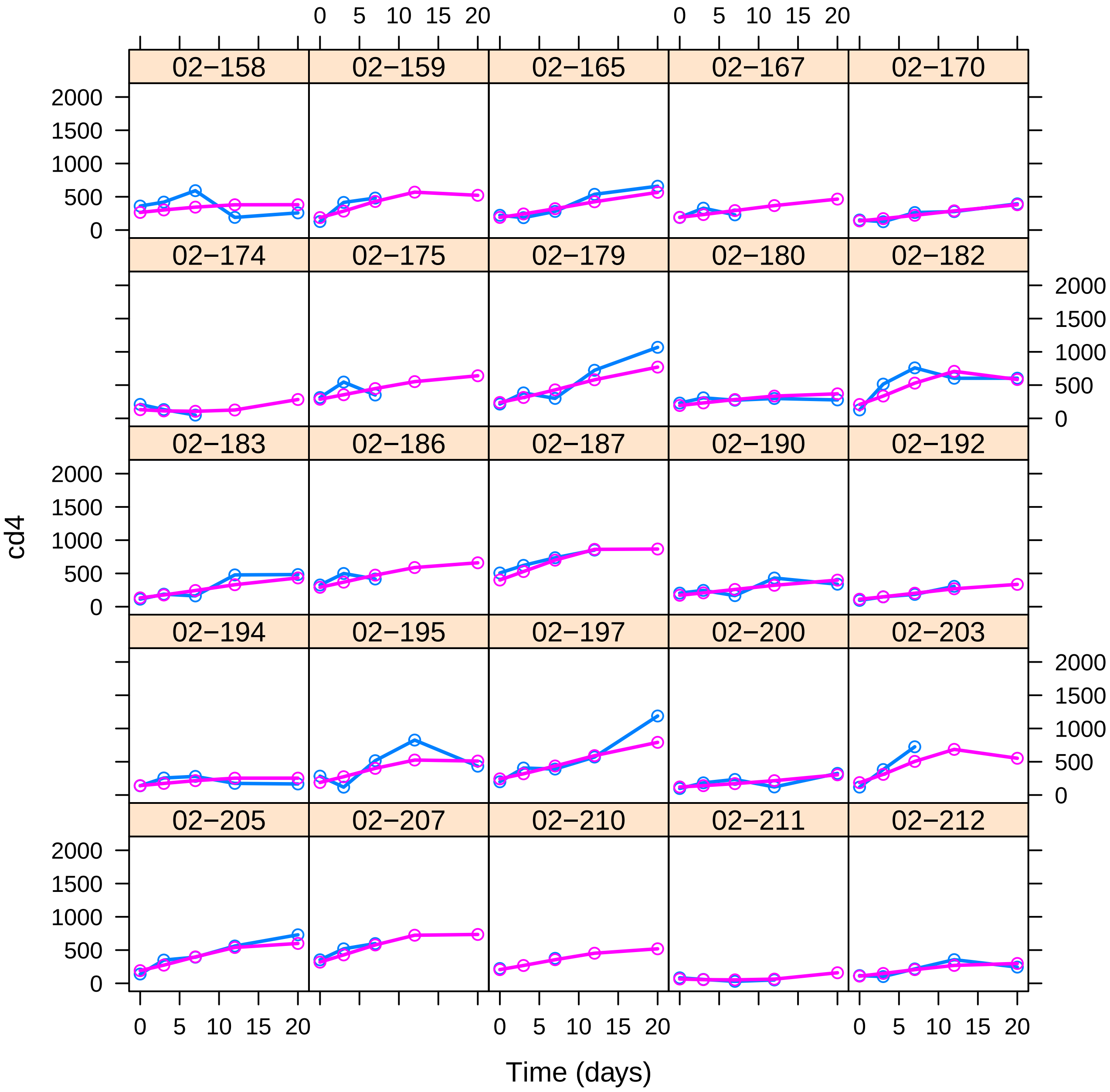

02−158
02−159
02−165
02−167
02−170
02−174
02−175
02−179
02−180
02−182
02−183
02−186
02−187
02−190
02−192
02−194
02−195
02−197
02−200
02−203
02−205
02−207
02−210
02−211
02−212
cd4
Time (days)

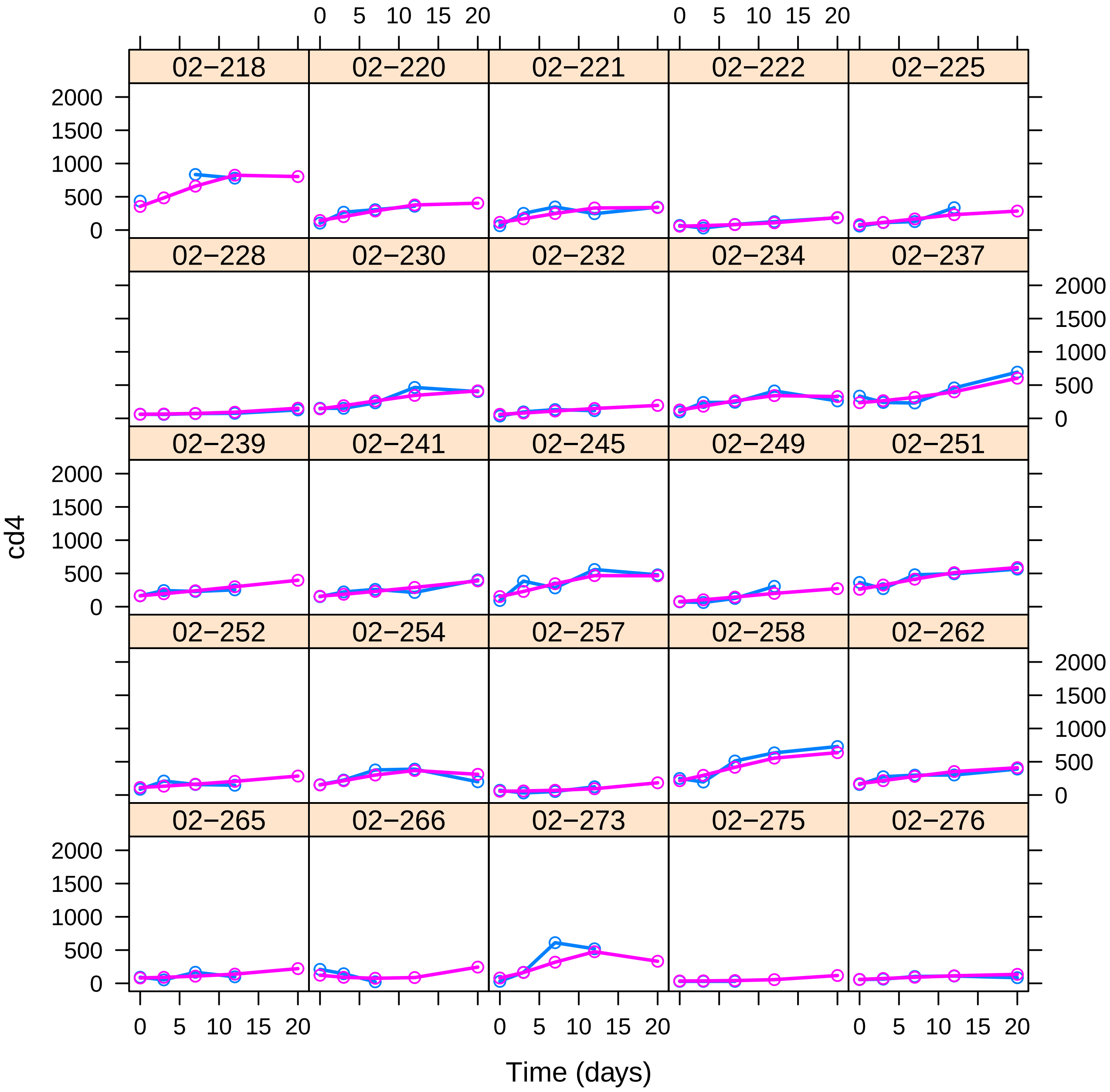
02−218
02−220
02−221
02−222
02−225
02−228
02−230
02−232
02−234
02−237
02−239
02−241
02−245
02−249
02−251
02−252
02−254
02−257
02−258
02−262
02−265
02−266
02−273
02−275
02−276
cd4
Time (days)

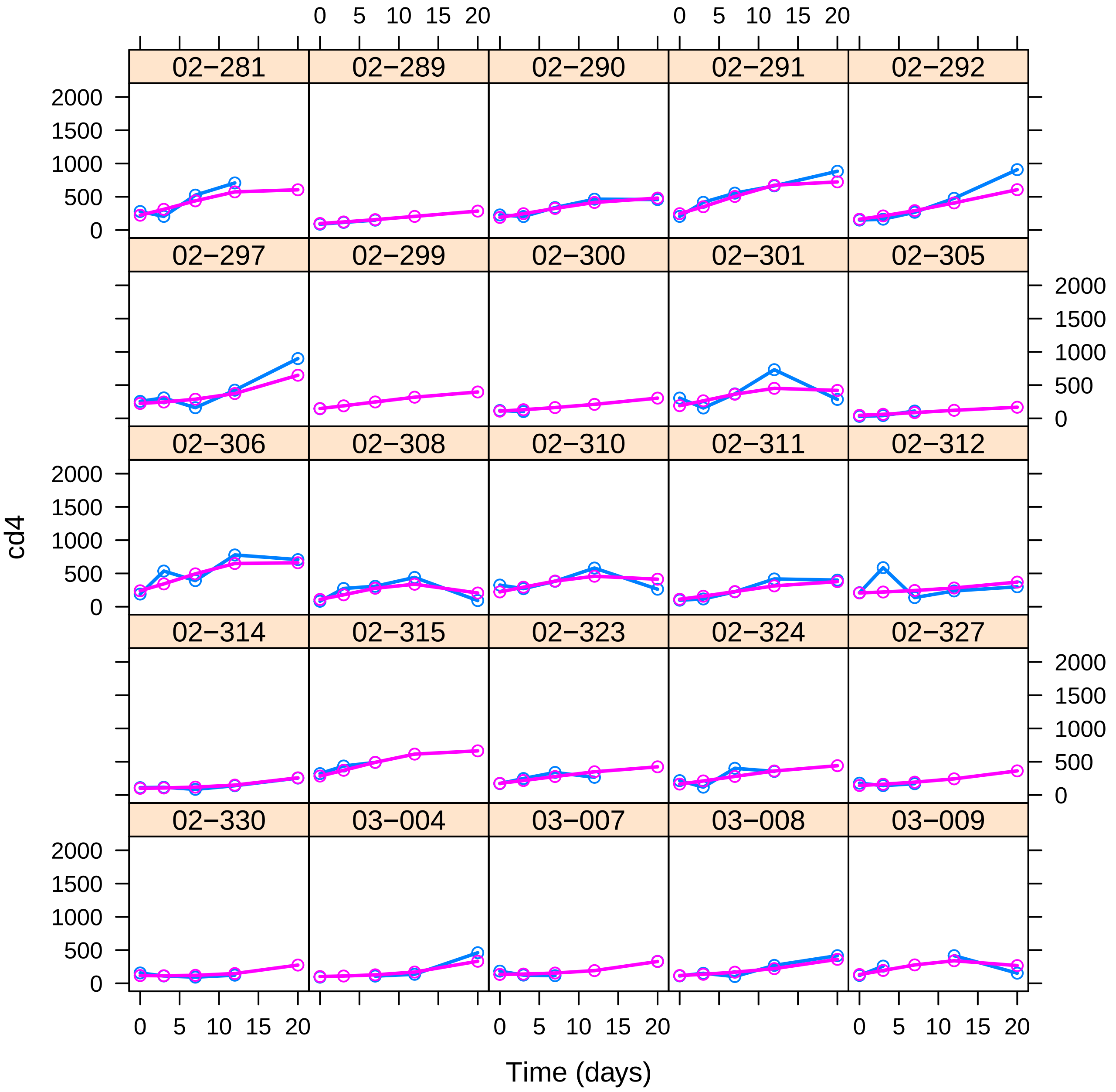
02−281
02−289
02−290
02−291
02−292
02−297
02−299
02−300
02−301
02−305
02−306
02−308
02−310
02−311
02−312
02−314
02−315
02−323
02−324
02−327
02−330
03−004
03−007
03−008
03−009
cd4
Time (days)

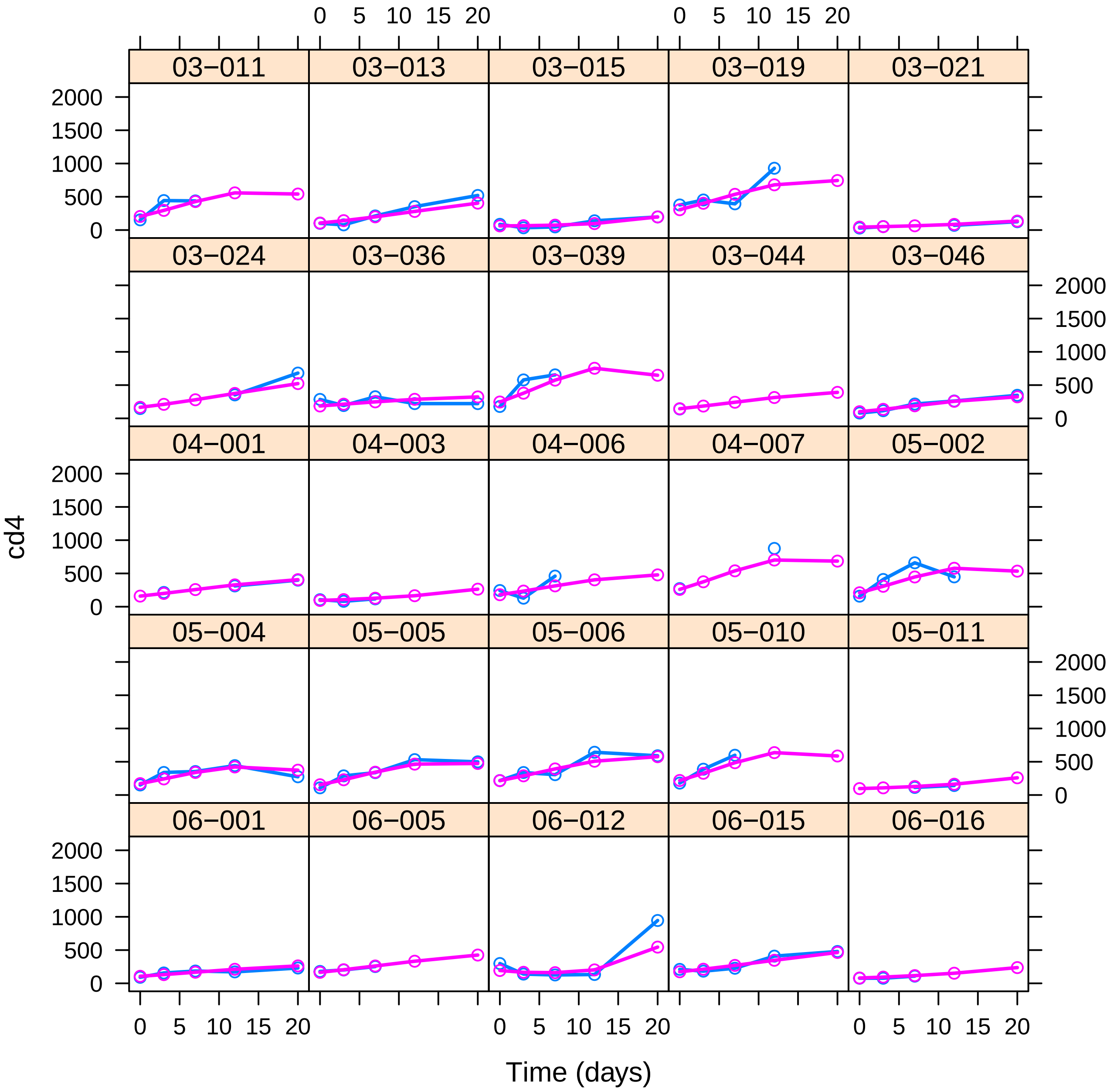

03−011
03−013
03−015
03−019
03−021
03−024
03−036
03−039
03−044
03−046
04−001
04−003
04−006
04−007
05−002
05−004
05−005
05−006
05−010
05−011
06−001
06−005
06−012
06−015
06−016
cd4
Time (days)
0
5
10
15
20
0
500
1000
1500
2000

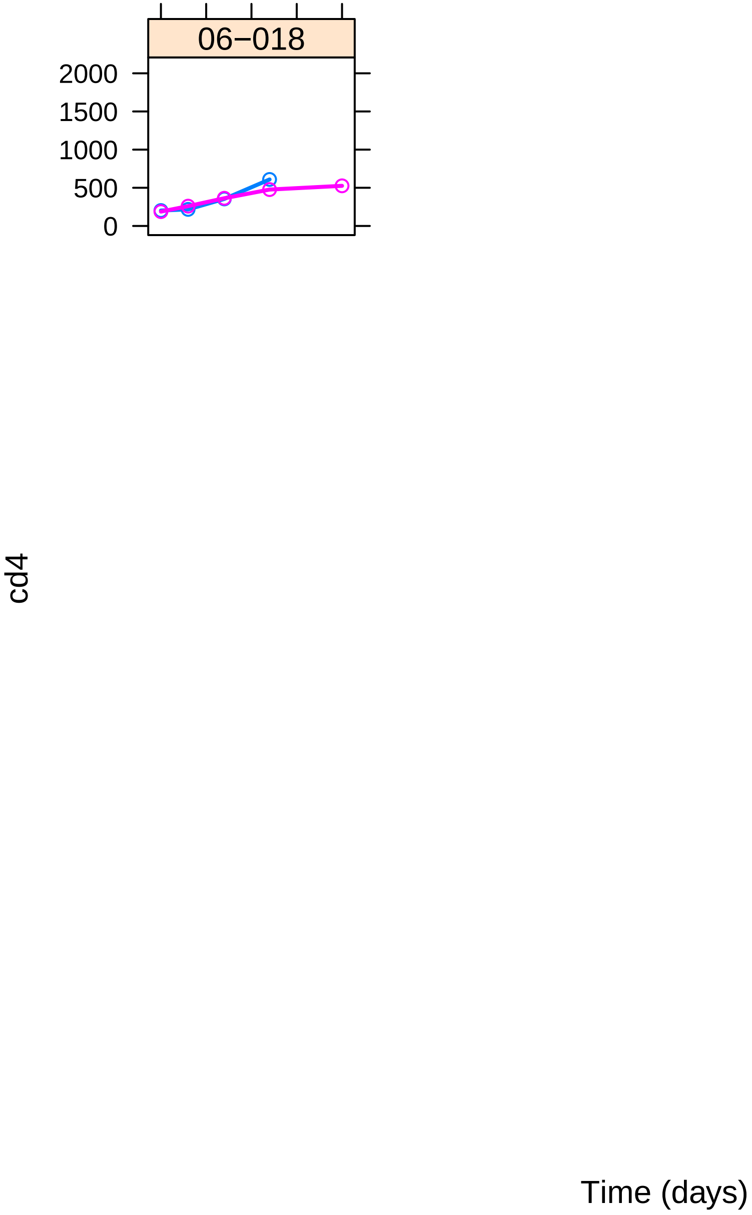
06−018
2000
1500
1000
500
0
cd4
Time (days)

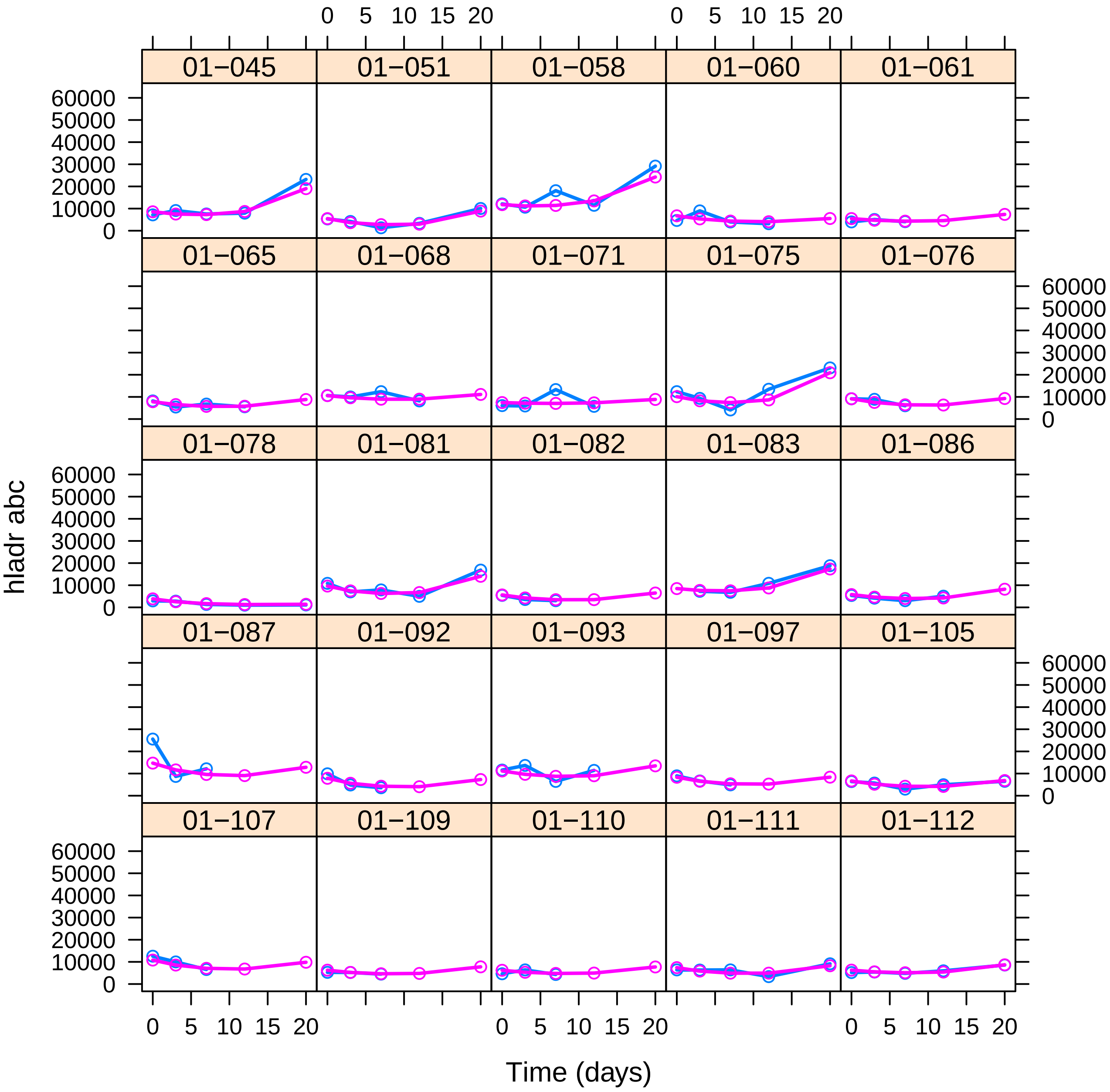

01−045
01−051
01−058
01−060
01−061
01−065
01−068
01−071
01−075
01−076
01−078
01−081
01−082
01−083
01−086
01−087
01−092
01−093
01−097
01−105
01−107
01−109
01−110
01−111
01−112
hladr abc
Time (days)
0 5 10 15 20
0 10000 20000 30000 40000 50000 60000

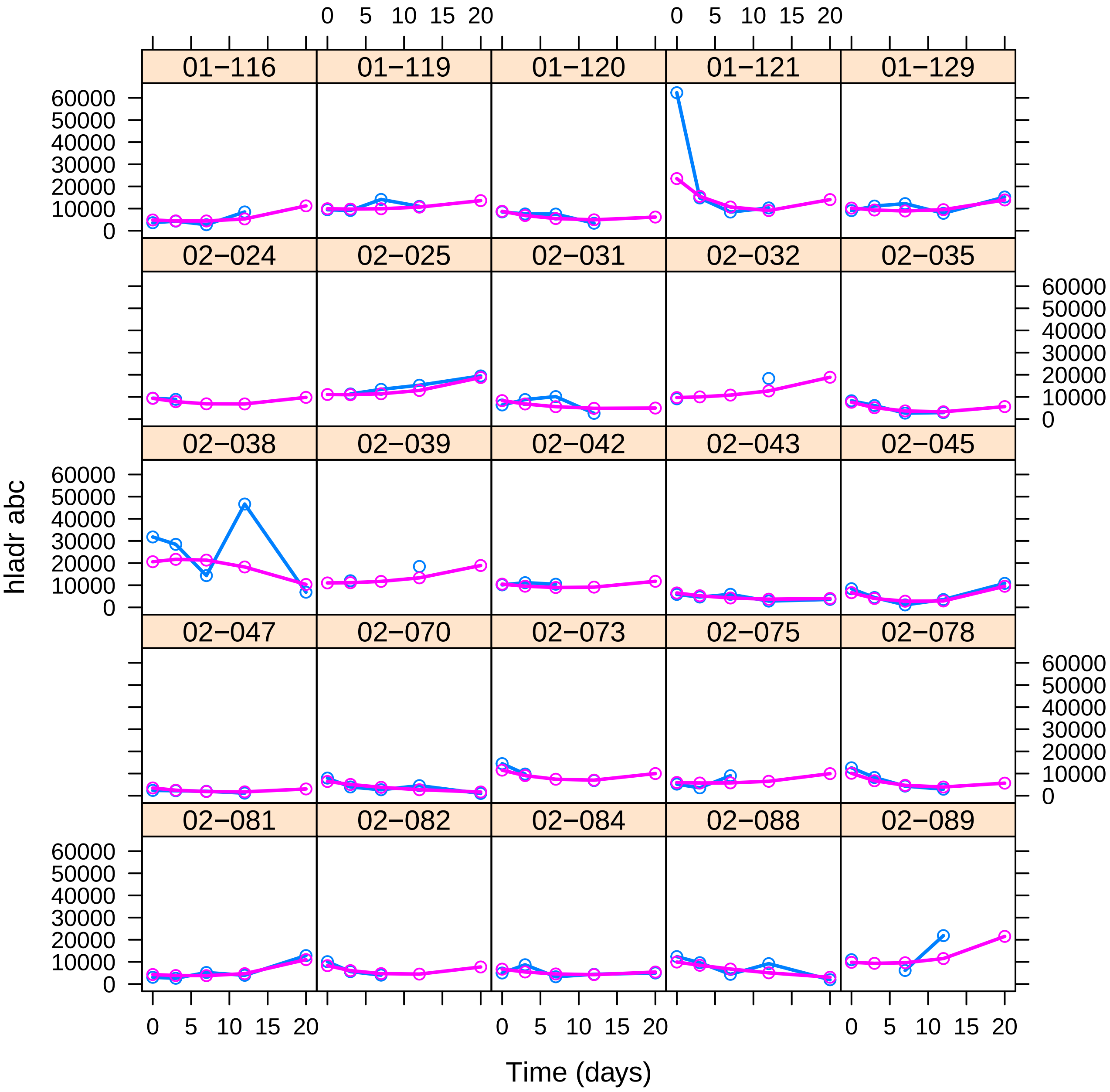

01−116
01−119
01−120
01−121
01−129
02−024
02−025
02−031
02−032
02−035
02−038
02−039
02−042
02−043
02−045
02−047
02−070
02−073
02−075
02−078
02−081
02−082
02−084
02−088
02−089
hladr abc
Time (days)
0
5
10
15
20
0
10000
20000
30000
40000
50000
60000

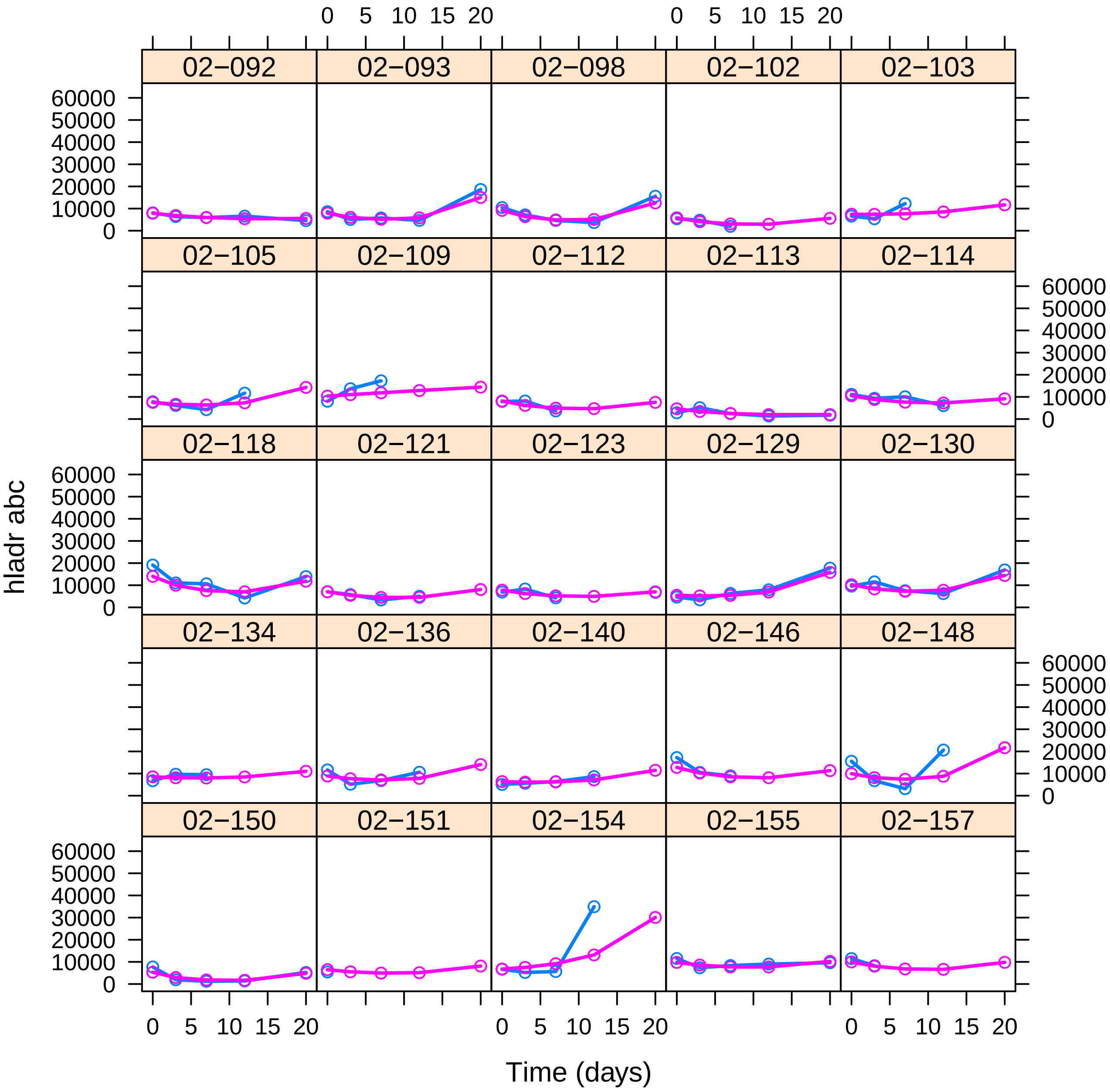
02−092
02−093
02−098
02−102
02−103
02−105
02−109
02−112
02−113
02−114
02−118
02−121
02−123
02−129
02−130
02−134
02−136
02−140
02−146
02−148
02−150
02−151
02−154
02−155
02−157
hladr abc
Time (days)
0
10000
20000
30000
40000
50000
60000
0
5
10
15
20

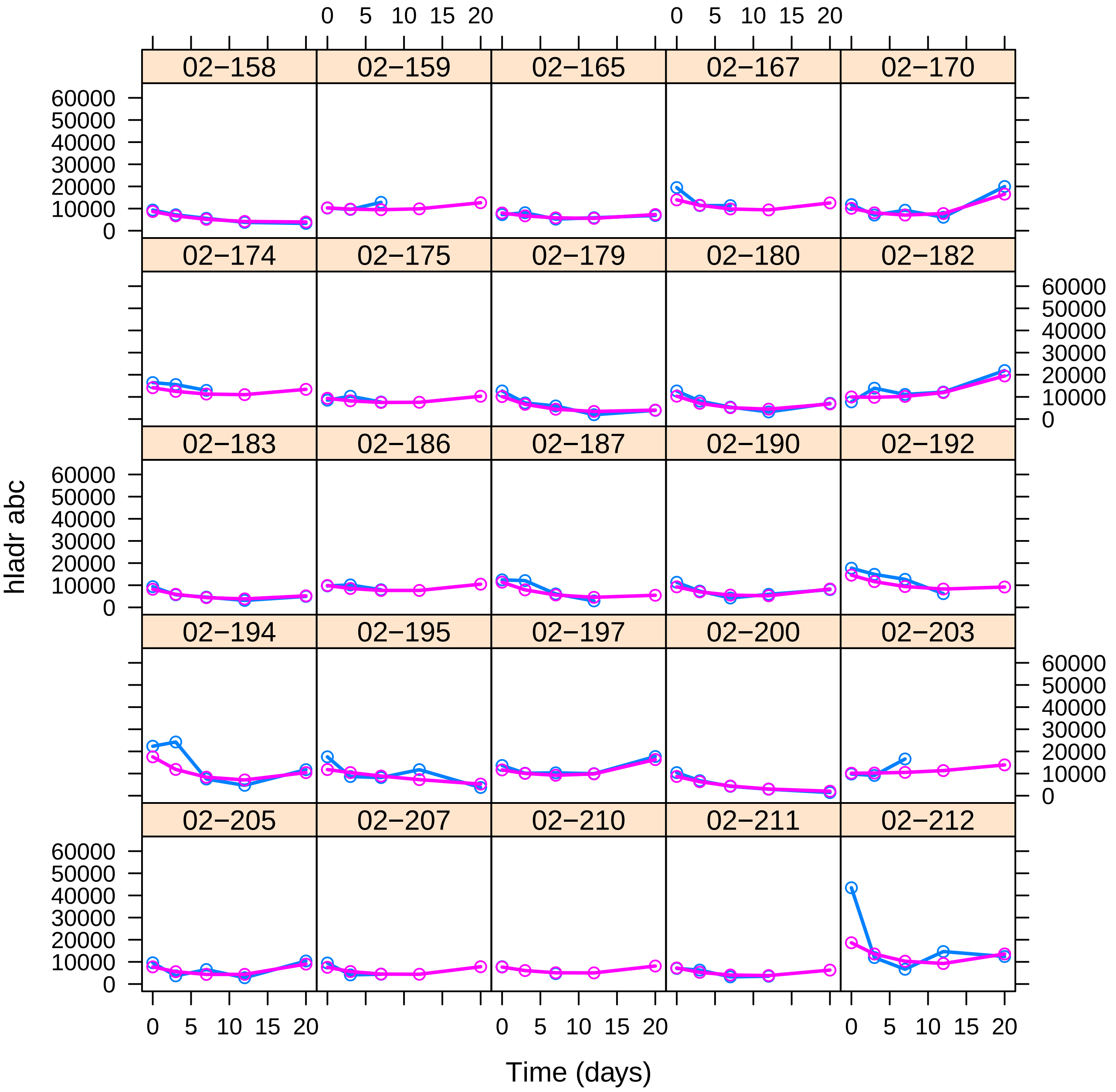
02−158
02−159
02−165
02−167
02−170
02−174
02−175
02−179
02−180
02−182
02−183
02−186
02−187
02−190
02−192
02−194
02−195
02−197
02−200
02−203
02−205
02−207
02−210
02−211
02−212
hladr abc
Time (days)
0
10000
20000
30000
40000
50000
60000
0
5
10
15
20

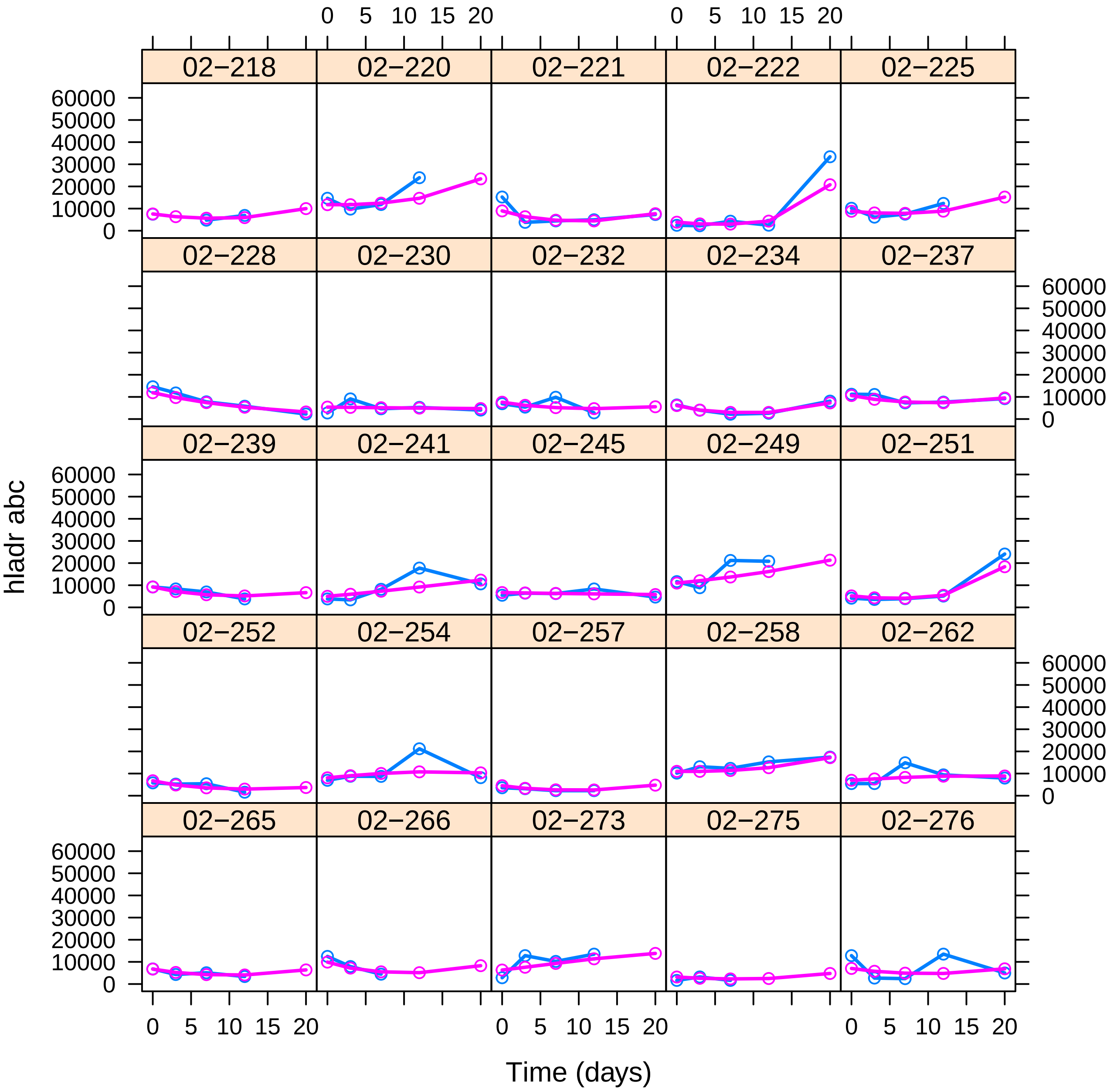

02−218
02−220
02−221
02−222
02−225
02−228
02−230
02−232
02−234
02−237
02−239
02−241
02−245
02−249
02−251
02−252
02−254
02−257
02−258
02−262
02−265
02−266
02−273
02−275
02−276
hladr abc
Time (days)
0 10000 20000 30000 40000 50000 60000
0 5 10 15 20

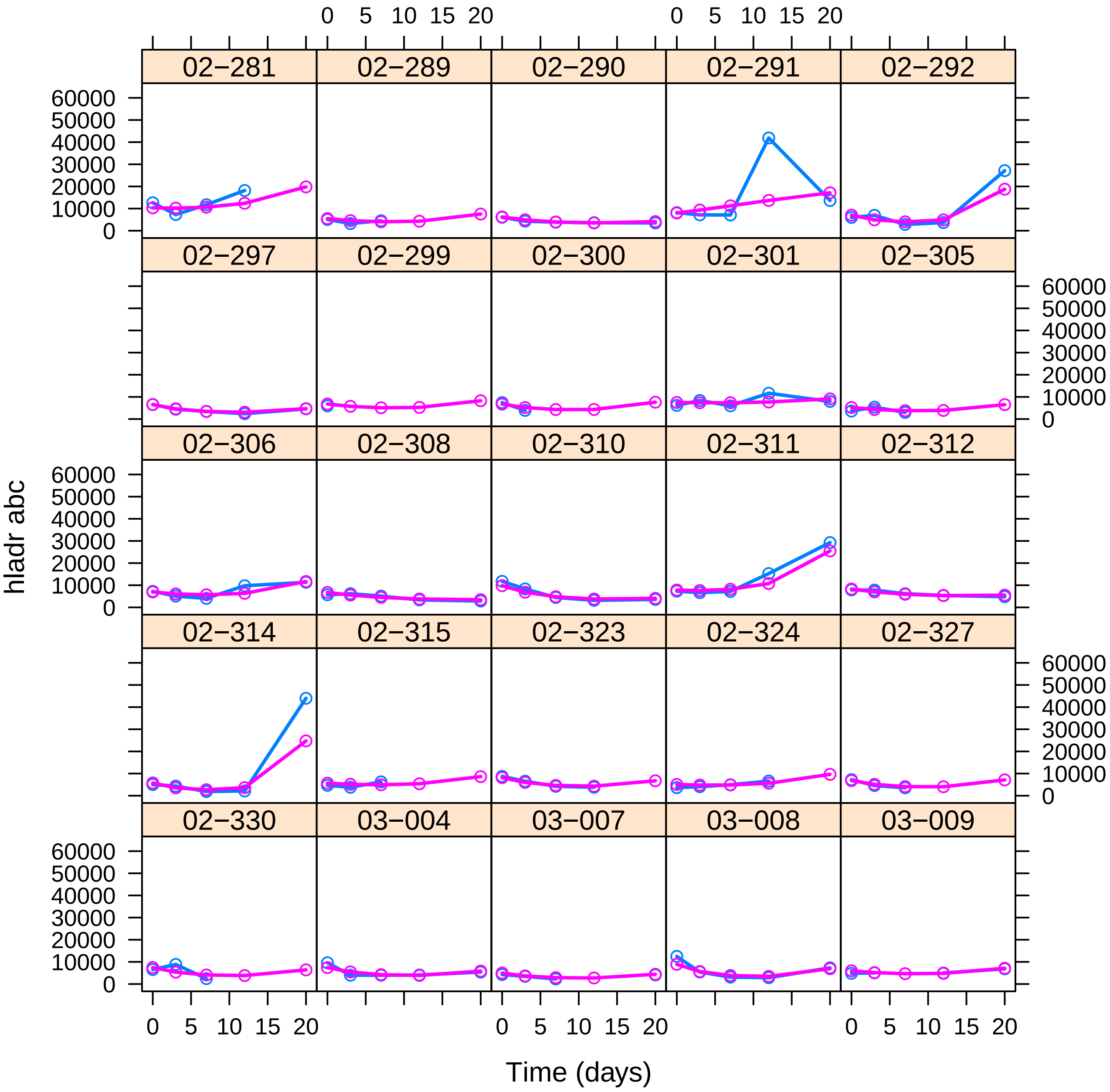
02−281
02−289
02−290
02−291
02−292
02−297
02−299
02−300
02−301
02−305
02−306
02−308
02−310
02−311
02−312
02−314
02−315
02−323
02−324
02−327
02−330
03−004
03−007
03−008
03−009
hladr abc
Time (days)
0
10000
20000
30000
40000
50000
60000
0
5
10
15
20

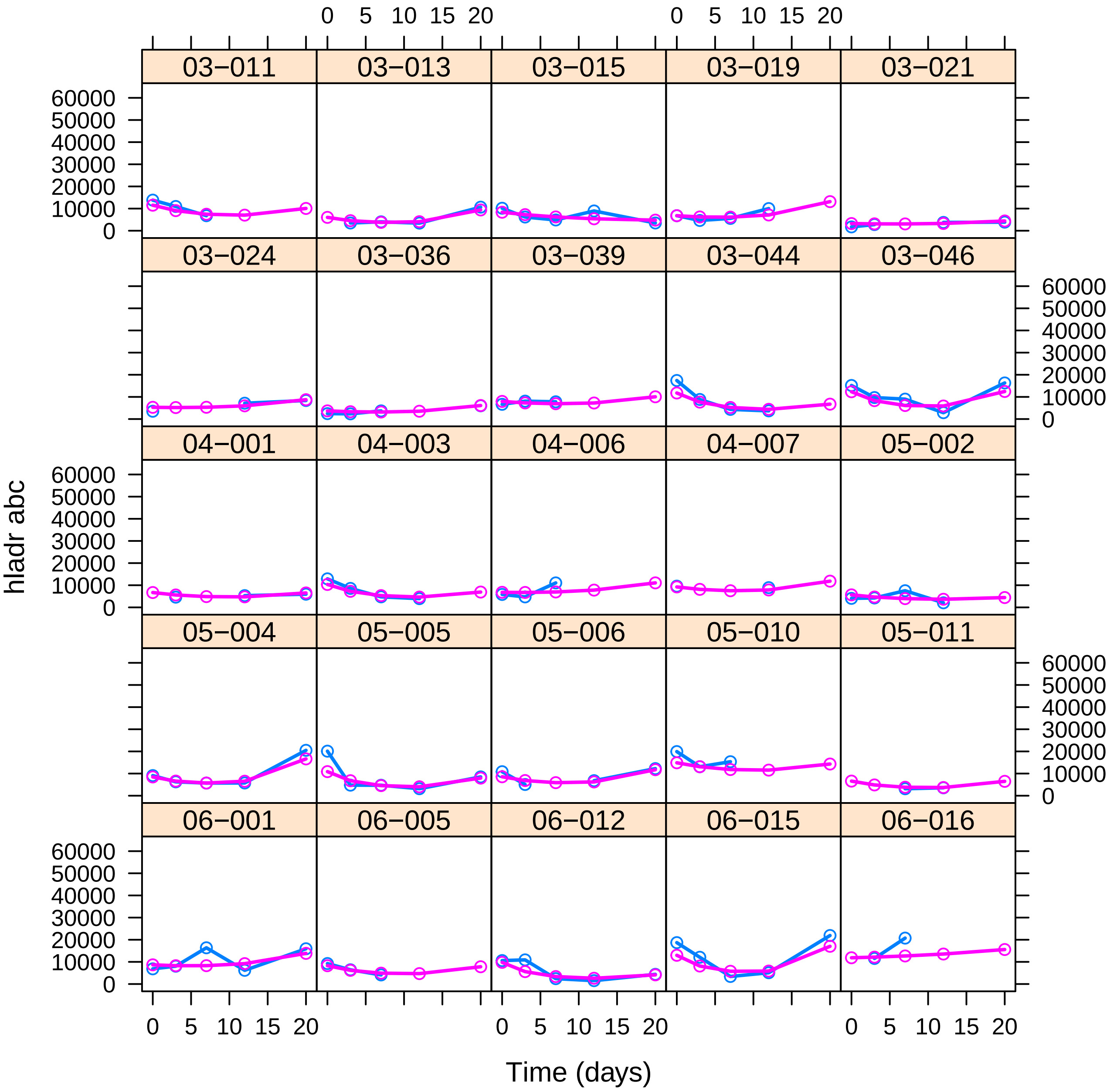
03−011
03−013
03−015
03−019
03−021
03−024
03−036
03−039
03−044
03−046
04−001
04−003
04−006
04−007
05−002
05−004
05−005
05−006
05−010
05−011
06−001
06−005
06−012
06−015
06−016
hladr abc
Time (days)
0 5 10 15 20
0 10000 20000 30000 40000 50000 60000

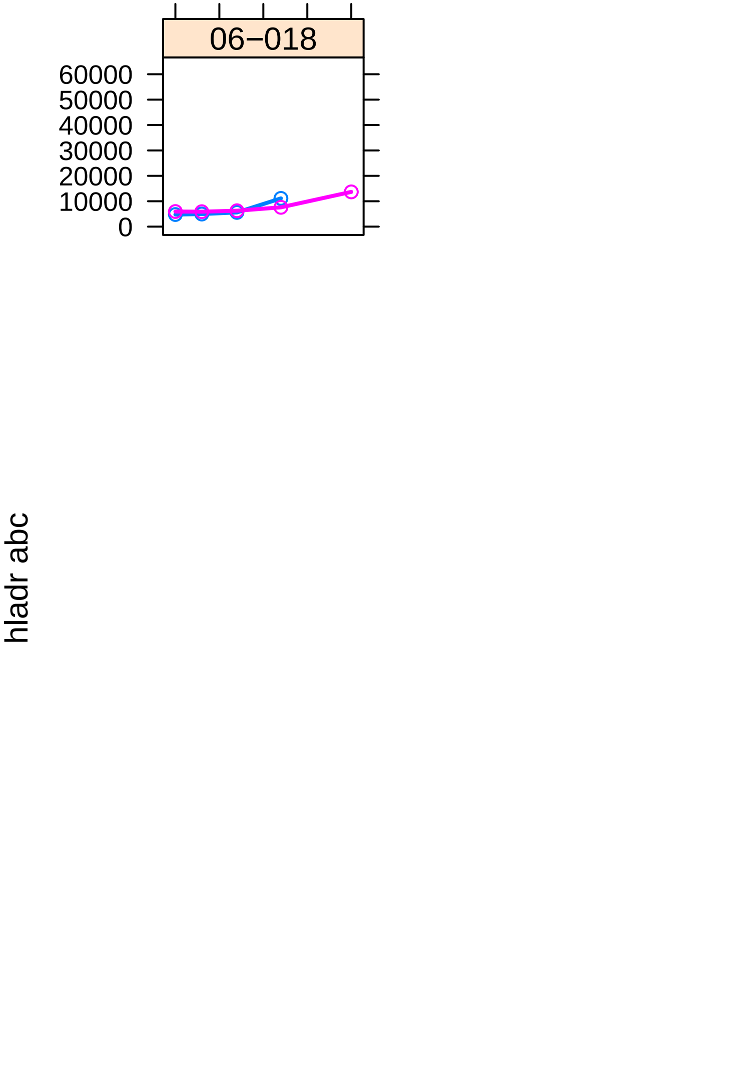
06−018
60000
50000
40000
30000
20000
10000
0
hladr abc
Time (days)
