# Supplementary material for: Monitoring monocyte HLA-DR expression and CD4 + T lymphocyte count in dexamethasone-treated severe COVID-19 patients
Source: Ann Intensive Care. 2024 May 18;14:76. doi: 10.1186/s13613-024-01310-5 (PMC11102415; doi:10.1186/s13613-024-01310-5)

Monitoring monocyte HLA-DR expression and CD4+ T lymphocyte count in dexamethasone-treated severe COVID-19 patients

ONLINE DATA SUPPLEMENT - Performances of the computation

The graphical analysis of the adequacy of the modeled and observed individual curves of HLA-DR and CD4 counts, provided in the Supplementary Information, demonstrated excellent agreement between the observed data and the model (**Figure S2 and S3**). Additionally, we calculated the correlation between observed and modeled HLA-DR and CD4 counts, as well as the percentage difference between observed and modeled values (see table below, and figures illustrating the distribution of the difference proportion between observed and modeled values). Given the high agreement between observed and modeled values at all time points, we concluded that it was reasonable to utilize the predictions of HLA-DR and CD4 counts at day 10 from the trajectory models.

	HLA_DR	CD4 counts
Correlation between observed and modelled values	0.866	0.938
% difference between observed and modelled value Median (Q1; Q3)	-0.04% (-1.88%; 1.78%)	0.40% (-3.02%; 3.10%)

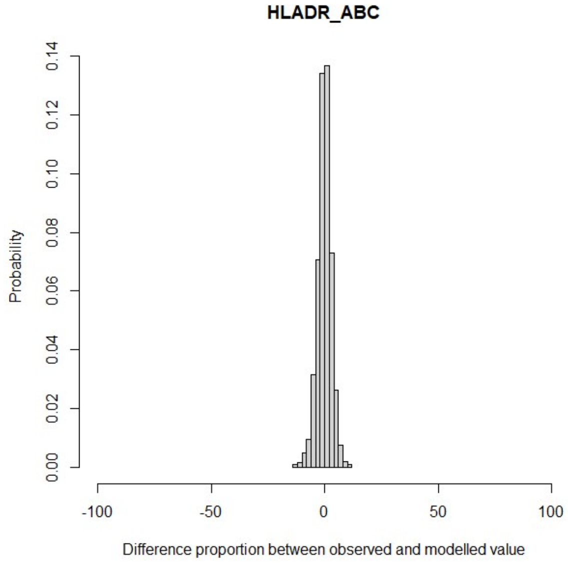

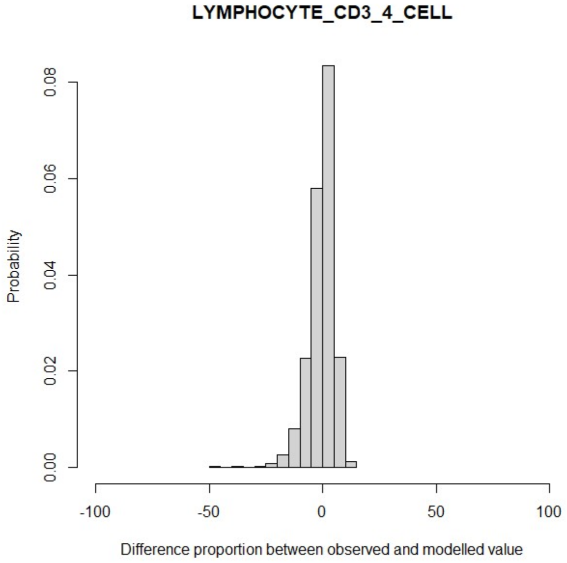

Supplement: Supplementary file 2 — Supplementary material 2. [file 13613_2024_1310_MOESM2_ESM.pdf]
